# Comparative genome analysis of human pathogen Parvimonas micra revealed strain JM503A as potential novel species in the genus Parvimonas and high intra-species functional diversity

**DOI:** 10.1099/mgen.0.001511

**Published:** 2025-09-22

**Authors:** Roja Suresh, Susanthika Jayachandiran, Pratebha Balu, Pajanivel Ranganadin, Ramasamy Dhamodharan

**Affiliations:** 1 Mahatma Gandhi Medical Advanced Research Institute (MGMARI), Sri Balaji Vidyapeeth (Deemed to be University), Pondicherry 607402, India; 2 Indira Gandhi Institute of Dental Sciences (IGIDS), Sri Balaji Vidyapeeth (Deemed to be University), Pondicherry 607402, India; 3 Mahatma Gandhi Medical College and Research Institute (MGMCRI), Sri Balaji Vidyapeeth (Deemed to be University), Pondicherry 607402, India

**Keywords:** antimicrobial resistance, comparative genomics, novel species, pan-genome analysis, *Parvimonas micra*, phylogenetic analysis, virulence factors

## Abstract

*Parvimonas micra* is a Gram-positive, anaerobic bacterium commonly found in the oral cavity, skin and gastrointestinal tract. While typically a harmless organism, it can cause infections in individuals with weakened immune systems, leading to conditions like periodontitis and deep-tissue abscesses. This study focuses on the comparative genomic analysis of *P. micra* to explore its evolutionary relationships, antimicrobial resistance profiles and functional diversity by assessing phylogenetic analyses, resistance genes, virulence factors, mobile genetic elements, carbohydrate-active enzymes and pan-genome analysis. Comparative genomic analysis of 11 *P. micra* strains reveals significant functional variations among the strains, indicating notable interspecies diversity. Phylogenetic and comparative genome analysis revealed that strain JM503A is taxonomically distinct from the *P. micra* species, with genome similarity ranging from 54% to 61%. The 16S rRNA sequence similarity of strain JM503A is 98.28%, indicating a distinct phylogenetic position. The average nucleotide identity value ranging from 91.32% to 91.7% and digital DNA–DNA hybridization values ranging from 43.00% to 44.00% of JM503A with other strains are below the cutoff values <95% and <70%, respectively, which confirms JM503A as a novel species. Based on its evolutionary relationships, strain JM503A is identified as a potential new species of *Parvimonas*, providing important evidence for its reclassification as a new species within the genus *Parvimonas*.

**Impact Statement**

*Parvimonas micra* plays a significant role in human health, particularly in oral and systemic infections, as it is often linked to chronic periodontitis, contributing to gum inflammation and tissue destruction. In immunocompromised individuals, *P. micra* can lead to more severe infections such as abscesses, endocarditis and osteomyelitis. *P. micra* can also lead to systemic infections by entering the bloodstream and forming biofilms that resist treatment. Recognizing proper taxonomic identification and functional diversity of this pathogen is essential for effective management of both oral and overall health. The dearth of comparative genomic studies on this species in existing literature motivated us to investigate the taxono-genomic and functional diversity of the currently available complete genome datasets of *P. micra*. Based on phylogenetic and comparative genomic analysis, we found a potential new species under the *Parvimonas* genus. We also found intra-species functional diversity with respect to antimicrobial resistance, virulence and adaptation-specific genes. Our finding of new *Parvimonas* species and comparative genomic analysis lays the foundation for future research into functional genomics, taxono-genomics, evolutionary studies and pathogenicity of *Parvimonas* species. Our findings are particularly important for researchers interested in studying, diagnosing and treating antimicrobial-resistant oral pathogens linked to systemic diseases. Publishing our findings in Microbial Genomics will reach global researchers and contribute to the field of taxono-genomics and comparative genomics for differential characterization of human pathogenic bacterial species.

## Data Summary

Genomic sequences used in this study were obtained from NCBI’s Genome database, and accession numbers for the genomes are provided in the manuscript. Please refer to Table S1 (available in the online Supplementary Material) for the list of genome sequences used and their corresponding assembly accession numbers. The data can also be accessed at https://www.ncbi.nlm.nih.gov/datasets/genome/?taxon=33033&assembly_level=3.

## Introduction

*Parvimonas micra* is a Gram-positive, anaerobic coccus commonly found in the oral cavity and gastrointestinal tract [1,2]. It was reclassified from the *Peptostreptococcus* and *Micromonas* genera in 2006 [3]. This oral pathobiont is associated with periodontal disease and systemic infections such as lung abscesses, spondylodiscitis, septic arthritis, prosthetic joint infections and bacteraemia, which can lead to serious complications like mycotic aneurysms [4,10]. *P. micra* is particularly known for causing monomicrobial infections related to spinal discitis and infective endocarditis [9,11]. Risk factors for infection include immunosuppression, joint inflammation, malignancy, diabetes, arthroplasty and dental procedures [1,8, 9]. *P. micra* often presents with back pain in case of spondylodiscitis and is typically treated with antibiotics, sometimes requiring surgical intervention for favourable outcomes [12]. However, the rise in antibiotic resistance in *P. micra* poses significant challenges for effective treatment [13].

The strains of *P. micra* have been isolated from dental and abdominal abscesses and are increasingly recognized as a potential biomarker for malignancies. It is also found in high numbers at sites of inflammatory dysbiosis and has been associated with various mucosal diseases and cancers [14]. The outer membrane vesicles of *P. micra* can activate inflammatory pathways in host cells, contributing to conditions like oral lichen planus by promoting the secretion of tumour necrosis factor-alpha [15]. The availability of genome sequences and genetic manipulation techniques opens new avenues for investigating the role of *P. micra* in polymicrobial infections and tumourigenesis [14]. Notably, *P. micra* exhibits natural competence, enabling the development of genetic tools for studying its pathobiology [16]. Genotypic diversity among *P. micra* isolates has been observed through 16S rRNA PCR-RFLP analysis, revealing two major clusters with multiple genotypes [4]. Historically difficult to culture, *P. micra* is now more frequently identified due to advanced techniques like MALDI-TOF-MS and 16S rRNA sequencing [1,13].

Recent studies have shown a concerning increase in *P. micra* resistance to antibiotics, particularly doxycycline and clindamycin, which raises concerns about their indiscriminate use in treating periodontitis [13,17]. While *P. micra* is still susceptible to amoxicillin and metronidazole, the emergence of antibiotic resistance underscores the importance of conducting susceptibility testing in all cases [13]. *P. micra* has been shown to trigger the production of gingipains, a virulence factor (VF) of *Porphyromonas gingivalis*, in multi-species communities, potentially contributing to the development of periodontitis [18]. Additionally, *P. micra* has been identified as one of the species that distinguishes refractory periodontitis from treatable periodontitis and periodontal health, indicating its significance in the progression and resistance to treatment of periodontal disease [19]. These findings underscore the importance of *P. micra* as a pathobiont in oral health and its potential role in driving disease progression.

Despite the growing recognition of *P. micra* as an opportunistic pathogen, critical research gaps persist. These include a limited understanding of its genomic diversity and evolutionary adaptations across different strains and environments, as well as insufficient studies exploring the functional roles of specific VFs and antibiotic resistance genes. Furthermore, the isolation and ecological differentiation of *P. micra* across various niches, particularly beyond the oral cavity, remain poorly understood. Addressing these gaps is essential for advancing diagnostic, preventive and therapeutic strategies against *P. micra*-associated infections. Additional phylogenomic studies are necessary to clarify the evolutionary relationships and divergence patterns among *P. micra* strains, with a focus on exploring the intra-species genomic diversity and potential functional differentiation within the species.

## Methods

### Genome annotation

The complete genomic sequences of 11 strains of *P. micra* were retrieved from the National Centre for Biotechnology Information (NCBI) (https://www.ncbi.nlm.nih.gov/genome) genomic database [20]. The genomic information of *P. micra* was summarized (Table S1). Comprehensive information on the isolation source, country of origin and associated diseases of each strain was retrieved from the NCBI database. To ensure consistency in genome annotations for comparative analysis, the complete genome sequences of 11 *P*. *micra* strains were annotated using the Rapid Annotation using Subsystems Technology (RAST) server (https://rast.nmpdr.org/) [21]. The genomic data, including GC content, genome size, coding sequences (CDSs), RNA genes and number of functional genes in subsystems, were obtained from the RAST server for structural and functional characterization of *P. micra* strains analysed in this study.

The mobile genetic elements (MGEs) were predicted using the RAST server. The number of phages and prophages for each subsystem group according to the System for Effective and Efficient Discovery (SEED) classification was determined for 11 strains of *P. micra*. The Pathosystems Resource Integration Center (PATRIC) database (http://www.patricbrc.org) [22] was used to predict the antimicrobial resistance (AMR) genes and their families of each strain of *P. micra*. The IslandViewer database, an integrated interface, was used for computational identification and visualization of genomic islands [23]. A heatmap of AMR genes and virulence gene distribution in 11 *P*. *micra* isolates was constructed along with the hierarchical clustering using the Morpheus tool (https://software.broadinstitute.org/morpheus) [24]. The Dbcan3 database was used for predicting the number of carbohydrate-active enzymes (CAZymes) across 11 *P*. *micra* strains [25]. The number of CAZymes belonging to each carbohydrate family was compared. To analyse orthology-based functional sequence annotation of *P. micra* strains, the EggNOG mapper tool from the Galaxy EU database (https://usegalaxy.eu/) [26] was used to predict the clusters of orthologous gene (COG) functional categories.

### 16S rRNA sequence similarity and phylogeny

The complete 16S rRNA gene sequences of *P. micra* annotated from the 11 genomes were used to calculate sequence similarity and phylogenetic analysis. The Basic Local Alignment Search Tool (nucleotide) (blastn) tool (https://blast.ncbi.nlm.nih.gov/Blast.cgi) [27] was used to find the percentage of 16S sequence similarity of *P. micra* strains. The multiple sequence alignment of 16S rRNA sequences were performed using MUltiple Sequence Comparison by Log-Expectation (muscle) implemented in the molecular evolutionary genetics analysis (mega) software [28]. The phylogenetic tree was constructed using the maximum likelihood method with 1,000 bootstrap replicates using mega software [29]. *Finegoldia magna* was used as an outgroup, which allowed more robust determination of the evolutionary relationships among *P. micra* strains, providing a reference point for accurate inference of the tree’s topology and enhancing the overall interpretability of the phylogenetic analysis [16]. The tree was visualized using FigTree (http://tree.bio.ed.ac.uk/software/figtree/) software version 1.4.4.

### Whole-genome phylogeny

In this study, genome evolutionary genealogy employing ensemble statistics (Gegenees) software version 3.1 [30] was utilized for multiple-genome alignments. Multiple alignments of *P. micra* genomes were generated using a fragment size of 200 nt, a step size of 100 parameters and blastn, which was optimized for highly similar sequences. The heat plot was constructed based on a fragmented alignment using blastn with settings 50/25. A distance matrix file exported from the Gegenees software was used to construct neighbour-joining phylogenetic trees using SplitsTree version 4.19.2 [31] and viewed using FigTree software.

### Average nucleotide identity-based phylogeny

The average nucleotide identity (ANI) values among the complete genomes of 11 *P*. *micra* strains were calculated using the OrthoANIu algorithm implemented in the EzBioCloud ANI calculator [32]. A heatmap was generated using the Morpheus tool to visualize the ANI-based clustering. The ANI values were converted into genetic distance, and an ANI-based phylogenetic tree was constructed using SplitsTree version 4.19.2 [31] with the neighbour joining method.

### DNA–DNA hybridization analysis

To determine the pairwise DNA–DNA Hybridization (DDH) values of *P. micra*, *in silico* genome-to-genome comparisons were conducted using the Genome-to-Genome Distance Calculator (GGDC) accessible at https://ggdc.dsmz.de/ [33]. The analysis reported values derived from formula 2, which calculates identities based on the high-scoring segment lengths, ensuring accurate measurements of genetic similarity. All bootstrap confidence intervals were maintained below 0.5% DDH, reinforcing the reliability of the results.

### Pan-genome analysis

The pan-genome analysis of the 11 *P*. *micra* strains was conducted using the Bacterial Pan Genome Analysis (BPGA) tool version 1.3 [34] with their FASTA-formatted protein sequences. The core genome of *P. micra* was constructed using USEARCH software version 11 [35] with 50% sequence homology truncation value. Tandem alignment of core gene families was performed using muscle software [28]. The Gnuplot software, version 4.6, was used to draw the pan-genome and core genome point map. Functional analysis of core gene families, accessory gene families and unique gene families was conducted using COG annotation.

## Results and discussion

### Genomic characteristics

The complete genomes of 11 *P*. *micra* strains, isolated from different geographic locations and sources, were identified (Table 1). Among these, seven strains (PM79KC-AC-2, PM79KC-G-1, PM89KC-G-1, PM89KC-G-2, PM89KC-AC-1, PM102KC-G-1 and PM114KC-AC-1) were isolated from patients with colorectal cancer (CRC) and gingival crevicular fluid (GCF) in Galicia, Spain. Some of these strains may originate from the same patient, but each was processed and deposited as an independent genome assembly. The strain JM503A was isolated from a dental abscess in Portland, USA, and represents a geographically and clinically distinct strain compared with the others. Strain KCOM 1037, isolated from a postoperative maxillary cyst associated with maxillary sinusitis in Gwangju, South Korea, further contributes to the clinical diversity of the dataset [36]. Additionally, strain KCOM 1535 was obtained from a periapical abscess of the oral cavity, also in South Korea.

**Table 1.** Genome dataset of 11 complete *P. micra* strains, detailing isolation source, country of origin and associated diseases, retrieved from the NCBI database

Sl no.	Assembly accession	Strain	Isolation source	Isolation country	Host disease
1	GCA_900637905.1	NCTC11808	na	na	na
2	GCA_027474925.1	PM79KC-AC-2	Colon adenocarcinoma	Spain: Galicia	Colorectal cancer
3	GCA_027475005.1	PM102KC-G-1	GCF	Spain: Galicia	Colorectal cancer
4	GCA_027474905.1	PM79KC-G-1	GCF	Spain: Galicia	Colorectal cancer
5	GCA_027474945.1	PM89KC-G-1	GCF	Spain: Galicia	Colorectal cancer
6	GCA_027474965.1	PM89KC-G-2	GCF	Spain: Galicia	Colorectal cancer
7	GCA_027474985.1	PM89KC-AC-1	Colon adenocarcinoma	Spain: Galicia	Colorectal cancer
8	GCA_003454775.1	KCOM 1037	Postoperative maxillary cyst	South Korea: Gwangju	Maxillary sinusitis
9	GCA_000800295.1	KCOM 1535; ChDC B708	Oral cavity	Korea: Gwangju	Periapical abscess
10	GCA_037482165.1	JM503A	Dental abscess	USA: Portland	na
11	GCA_027475025.1	PM114KC-AC-1	Colon adenocarcinoma	Spain: Galicia	Colorectal cancer

NA - not available

Detailed genomic information, such as GC content, genomic size, CDSs, subsystems and RNAs, obtained from the RAST server is summarized in Table 2. The genome size of *P. micra* varies significantly among strains, with strain PM114KC-AC-1 having a smaller genome size of 1,532,745 bp, while strain PM79KC-AC-2 has a larger genome size of 1,755,474 bp. This variability in the species reflects strain-specific differences that can affect factors like pathogenicity, resistance traits and metabolic capabilities [37]. The data obtained from the RAST server revealed that the GC content of *P. micra* ranged from 27.9 mol% to 29.1 mol%. The strain PM79KC-AC-2 has a GC content of 29.1 mol%, which is the highest among the 11 strains compared. Notably, strain JM503A showed a distinct and lowest GC content of 27.9 mol% compared with the other strains. The CDSs also varied from 1,413 in strain PM114KC-AC-1 to 1,590 in PM79KC-AC-2. The number of RNA sequences varied from 47 to 51, including various RNA types such as tRNA and rRNA (Table S2), with strain KCOM 1037 having the highest count at 51. The tRNA genes ranged from 40 to 43, with most strains having 43. The rRNA gene count was consistently 10, except in NCTC11808 (12 copies) and KCOM 1,037 (15 copies). A single transfer-messenger RNA was present in all strains except JM503A. Non-coding RNA genes were mostly two per genome, but JM503A had one, and NCTC11808 had none. The number of functional subsystems, which represent different biological roles performed by gene groups, ranged from 153 (in strains PM89KC-G-1 and PM89KC-G-2) to 158 (in strains NCTC11808, PM102KC-G-1 and PM79KC-G-1). The analysis of *P. micra* genomes reveals intraspecific variability in both subsystems and genetic content among different strains of *P. micra,* which can translocate from the oral cavity to colorectal adenocarcinoma [38].

**Table 2.** The genomic information of all complete *P. micra* strains, detailing GC content, genome size, number of subsystems, number of CDSs and number of RNAs, retrieved from the RAST server. The strain JM503A exhibited a notably different GC content compared with other strains, which was highlighted in pink colour.

Sl no.	Assembly accession	Strain	GC content (mol%)	Genome size (bp)	CDS	Subsystem	RNA
1	GCA_900637905.1	NCTC11808	28.5	1,677,398	1,574	158	47
2	GCA_027474925.1	PM79KC-AC-2	29.1	1,755,474	1,590	156	47
3	GCA_027475005.1	PM102KC-G-1	29	1,733,605	1,585	158	49
4	GCA_027474905.1	PM79KC-G-1	28.8	1,678,242	1,537	158	47
5	GCA_027474945.1	PM89KC-G-1	28.8	1,676,768	1,560	153	49
6	GCA_027474965.1	PM89KC-G-2	28.8	1,676,568	1,559	153	49
7	GCA_027474985.1	PM89KC-AC-1	28.9	1,663,572	1,551	154	49
8	GCA_003454775.1	KCOM 1037	28.9	1,661,863	1,532	157	51
9	GCA_000800295.1	KCOM 1535; ChDC B708	28.6	1,627,009	1,496	157	48
10	GCA_037482165.1	JM503A	**27.9**	1,549,037	1,447	157	48
11	GCA_027475025.1	PM114KC-AC-1	28.4	1,532,745	1,413	155	49

### Phenotypic characteristics

All 11 *P*. *micra* strains demonstrated the same phenotypic properties, being characterized as Gram-positive cocci that are non-motile, non-sporing and ~0.3–0.7 µm long, dividing in pairs or short chains [39,40] (Table S3). All strains were obligate anaerobes, and most exhibited negative or unreported catalase, oxidase, indole, urease and saccharolytic activity. Generally, the optimal growth temperature was ~37 °C (only reported for KCOM 1535) from the PATRIC database. The main fermentation product was acetate, with small amounts of lactate and succinate [39]. Strain JM503A was the only strain to exhibit natural competence, highlighting strain-specific differences in the capacity for horizontal gene transfer [14].

### Phylogenetic characterization

#### 16S rRNA phylogeny

**Fig. 1.**
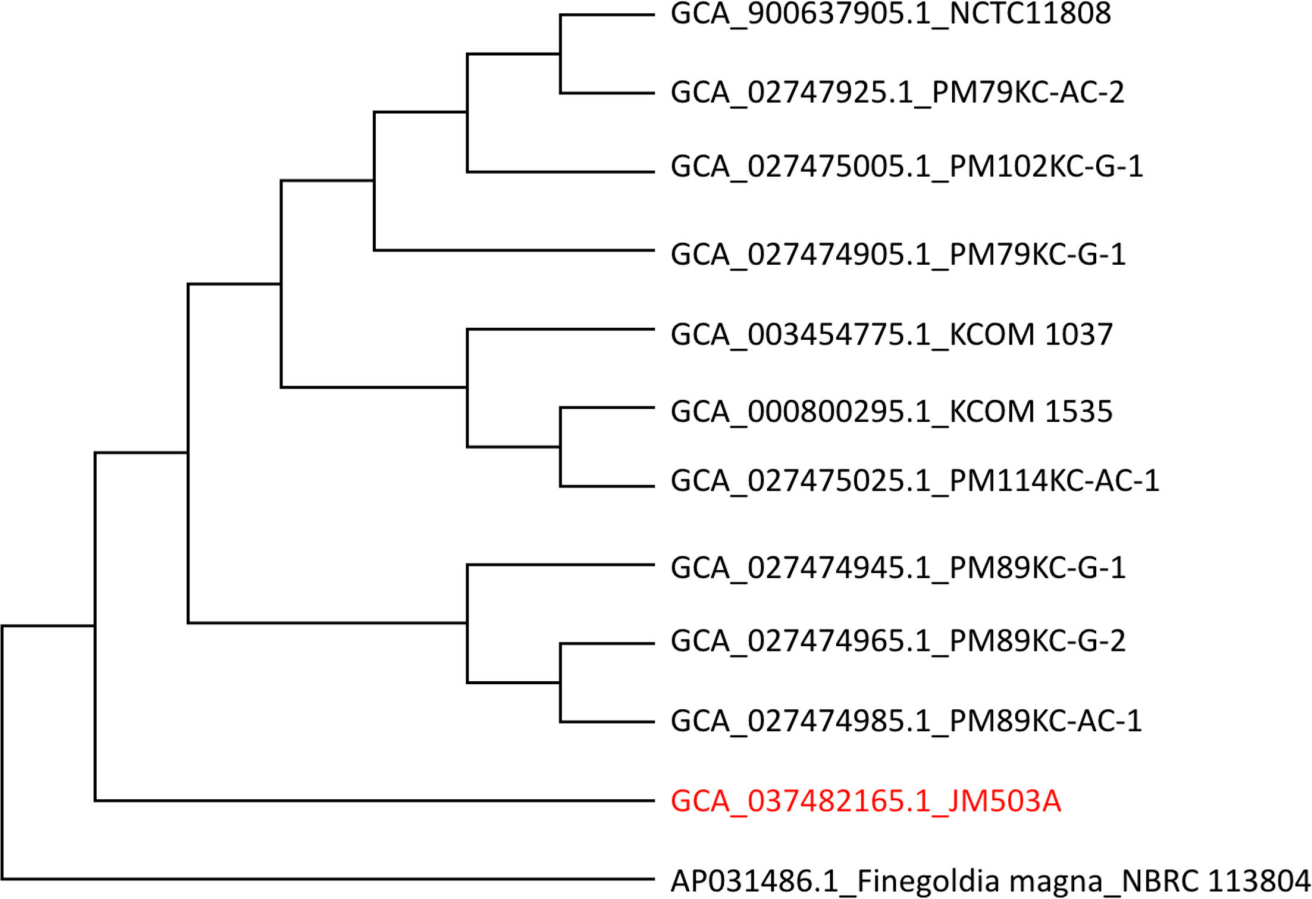
Phylogenetic analysis based on the 11 complete 16S rRNA gene sequences of *P. micra* showing 3 clusters, and the tree was constructed using the maximum likelihood method, based on muscle aligned sequences with 1,000 bootstrap replicates. *F. magna*, a close relative of *P. micra*, was used as an outgroup for rooting the phylogenetic tree.

The phylogenetic analysis using 16S rRNA gene sequences of 11 *P*. *micra* strains revealed 3 distinct clusters, as depicted in Fig. 1. Cluster 1 included strain PM89KC-G-1, PM89KC-G-2 and PM89KC-AC-1, while cluster 2 comprised the remaining strains. Strain JM503A formed a distinct lineage, indicating a divergent evolutionary relationship from the other ten strains of *P. micra. F. magna*, a closely related species to *P. micra* [16], was used as an outgroup. This analysis shows an intra-species variation within *P. micra* and enhances the understanding of its evolutionary relationships.

### Whole-genome phylogeny

**Fig. 2.**
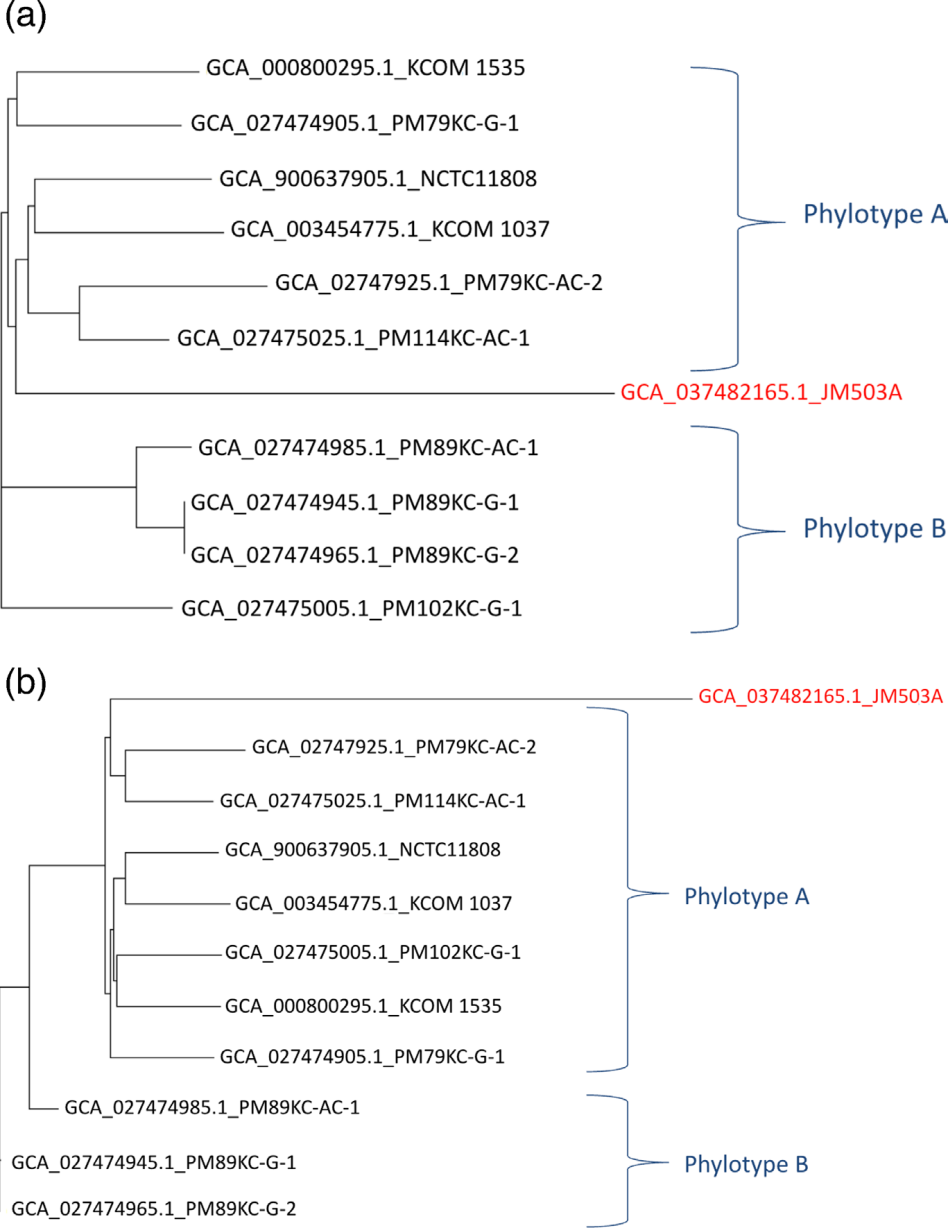
(**a**) Whole-genome-based phylogenetic tree of 11 *P*. *micra* strains. The phylogeny revealed two major phylotypes: phylotype A (including strains from multiple clinical sources) and phylotype B (comprising exclusively strains isolated from CRC patients). Strain JM503A is positioned separately, highlighted in red colour, forming a distinct lineage divergent from both phylotypes. (**b**) ANI-based phylogenetic tree of *P. micra* strains. The tree reveals two main phylotypes: Phylotype A (strains from diverse clinical sources) and phylotype B (strains isolated exclusively from CRC patients). Strain JM503A, highlighted in red colour, forms a distinct lineage separate from both phylotypes, consistent with its divergent genomic characteristics.

The Gegenees phylogenomic heatplot provides an overview of the genomic similarity and divergence among *P. micra* strains (Fig. S1). In the heatplot, strain JM503A consistently shows lower similarity scores ranging from 54% to 61% with the other ten strains, reinforcing its unique genetic divergence as observed in the 16S rRNA-based phylogenetic tree. The other strains clustered more closely, indicating higher levels of genomic similarity within the group of the remaining ten *P*. *micra* strains. Whole-genome phylogenetic analysis (Fig. 2a) of the 11 *P*. *micra* strains revealed a clear subdivision into 2 major phylotypes, designated as phylotype A and phylotype B, along with a distinct lineage represented by JM503A. Phylotype A comprised strains NCTC11808, PM79KC-G-1, KCOM1037, PM79KC-AC-2, PM114KC-AC-1 and KCOM1535, which were isolated from a variety of clinical sources, including both CRC and non-CRC patients, suggesting no specific clustering based on isolation source. In contrast, phylotype B included strains PM89KC-G-1, PM89KC-G-2, PM89KC-AC-1 and PM102KC-G-1, all exclusively isolated from GCF samples of CRC patients [38,41], indicating a possible clinical source-associated subgrouping within this lineage. Importantly, strain JM503A was placed separately from both phylotypes, forming a distinct lineage consistent with its observed genomic divergence, supported by ANI, DDH and 16S rRNA gene analyses, suggesting its possible classification as a novel species within the genus *Parvimonas*.

To further validate the taxonomic placement of strain JM503A, we performed both 16S rRNA gene and whole-genome blast analyses. The 16S rRNA gene analysis matched closely to the partial sequences of the *Parvimonas* oral taxa; no highly similar full-length 16S rRNA sequences corresponding to complete *P. micra* genomes were found (Table S4). The whole-genome blast comparisons indicated that the ANI of JM503A ranged from 93.95% to 95.19% when compared with other complete *P. micra* genomes (Table S5). This level of genomic similarity shows near or slightly below the accepted species delineation (>95–96%) [42], indicating that JM503A represents a unique taxonomic unit either within or closely associated with the *Parvimonas* genus. The level of genomic divergence may also reflect strain-specific or environmentally driven adaptations.

### ANI phylogeny

The ANI values among 11 *P*. *micra* strains were visualized in a heatplot (Fig. S2) representing the genomic similarities between pairs of strains, with different colours indicating the degree of similarity. Strain JM503A consistently shows lower similarity scores, with ANI values below 95%, compared with the other ten strains. This suggests significant genomic divergence in JM503A, setting it apart from the other strains. The higher ANI values of the remaining ten strains indicate their closer genetic relationships among them, while the lower ANI value of JM503A from other strains highlights its genetic diversity within *P. micra* [43]. The ANI-based phylogenetic reconstruction (Fig. 2b) had a strikingly similar clustering pattern as the whole-genome phylogenetic tree, demonstrating that the genomic relationship between *P. micra* strains was consistently supported by taxonomic clades. In the ANI phylogenetic tree, two primary phylotypes were observed, phylotype A and phylotype B, similar to the whole-genome tree. Phylotype A (PM79KC-AC-2, PM114KC-AC-1, NCTC11808, KCOM1037, PM102KC-G-1, KCOM1535 and PM79KC-G-1 in the analysis) included strains from patients with both CRC and non-CRC. Phylotype B (PM89KC-AC-1, PM89KC-G-1 and PM89KC-G-2 in analysis) only included strains derived from CRC patients [41], suggesting that it could represent a disease-associated subgroup. As before, JM503A formed a distinct lineage from both phylotype A and phylotype B, supporting the classification of JM503A as a novel species in the genus *Parvimonas*.

### Digital DDH analysis

The digital DDH (dDDH) analysis (Fig. S3) identified that all paired comparisons between 11 *P*. *micra* strains had dDDH values between 69.5% and 76.9%, either at or slightly below the commonly accepted species boundary of 70% for species delineation, which are indicative of intra-species genomic variation [42]. It should be noted that some pairs, such as PM102KC-G-1 vs. PM79KC-G-1 (75.8%), PM79KC-AC-2 vs. PM114KC-AC-1 (76.9%) and PM89KC-G-1 vs. PM89KC-G-2 (100%), show a higher level of similarity and have been identified as belonging to the same species. Other strains, such as KCOM 1037, KCOM 1535 and NCTC11808, exhibited moderate dDDH similarity (72.7%–76.9%) when compared with the rest of the dataset. Strain JM503A, on the other hand, shows the lowest level of dDDH values when compared with all other strains, ranging from 43.3% to 44.0%, which is significantly below the 70% species delineation threshold [42], strongly supporting that JM503A is a distinct taxonomic lineage outside of the *P. micra* species.

### Comparison of AMR genes

**Fig. 3.**
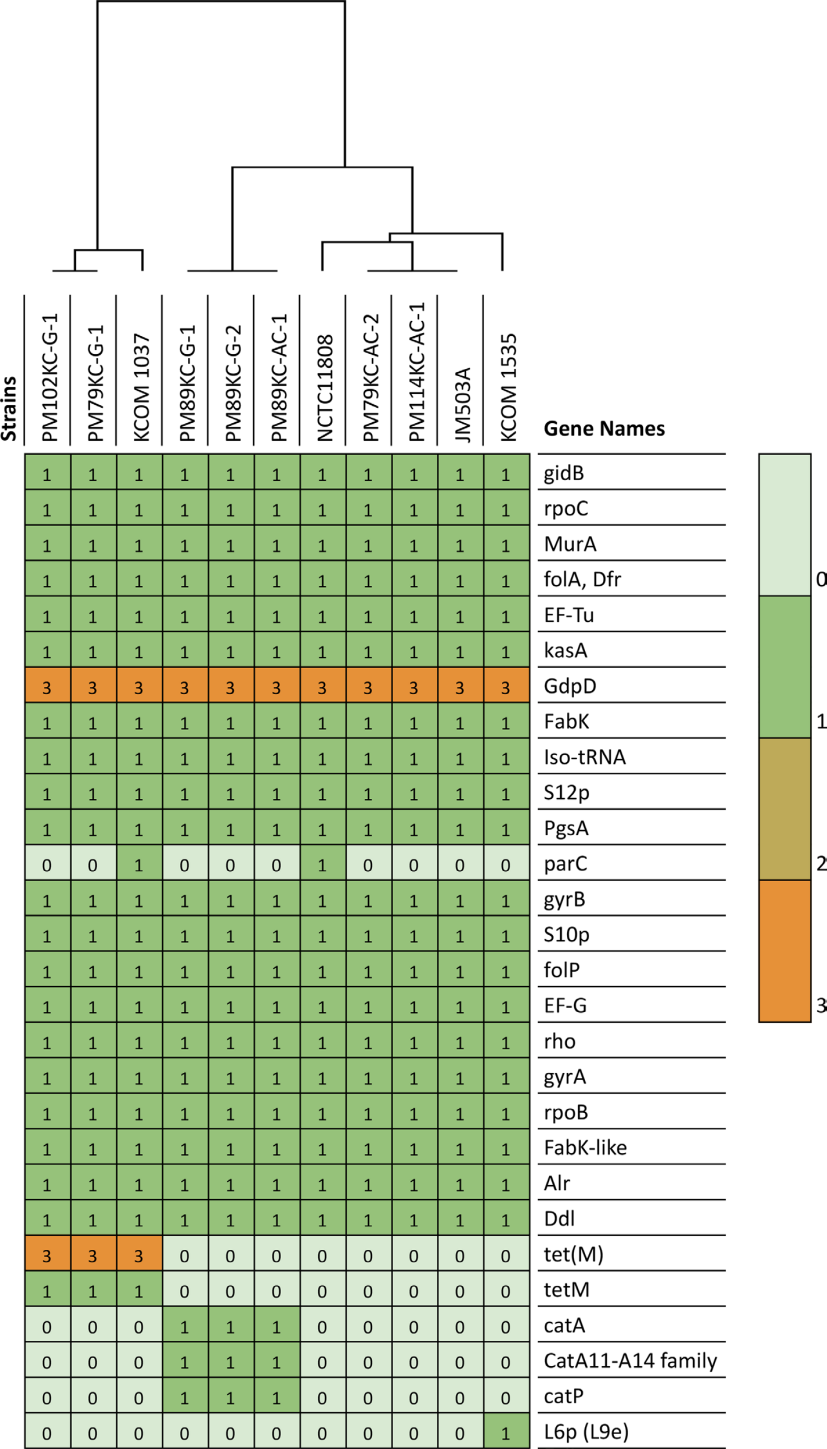
Heatmap of AMR gene distribution in 11 *P*. *micra* isolates. A total of 28 accessory AMR genes were identified by the PATRIC database in all 11 strains. The copy number, ranging from 0 to 3, was indicated by light green to orange. The hierarchical clustering showing the presence of strain-level AMR gene variation in the isolates was presented as a cladogram in the panel along the x-axis.

A total of 28 accessory AMR genes were identified using the PATRIC database, with gene copy numbers ranging from 0 to 3, and a heatmap visualizing the distribution of AMR genes across 11 *P*. *micra* isolates is shown in Fig. 3. The AMR profile includes genes such as gidB, rpoC, MurA, folA, Dfr, EF-Tu, kasA, GdpD, FabK, Iso-tRNA, S12p, PgsA, parC, gyrB, S10p, folP, EF-G, rho, gyrA, rpoB, FabK-like, Alr, Ddl, tet(M), tetM, catA, CatA11-A14 family, catP and L6p (L9e). The hierarchical clustering on the x-axis of the heatmap, shown as a cladogram, reveals significant variations in AMR gene presence at the strain level, highlighting the diversity in AMR profiles among the isolates. The GdpD gene, involved in glycerol metabolism, is uniformly distributed across all strains with a higher copy number of 3, potentially reflecting metabolic adaptations that could indirectly influence resistance mechanisms [44]. The presence of the ParC gene exclusively in strains NCTC11808 and KCOM 1037 is noteworthy as it encodes a subunit of DNA topoisomerase IV critical for bacterial DNA replication and is associated with quinolone resistance [45]. The L6p (L9e) gene, detected only in strain KCOM 1535 (ChDC B708), is related to ribosomal protein function and may contribute to ribosomal protection mechanisms against antibiotics targeting the ribosome [46]. The tet(M) gene, found in three strains, PM102KC-G-1, PM79KC-G-1 and KCOM 1037, with a copy number of 3, confers resistance to tetracyclines by encoding a ribosomal protection protein [47]. Genes such as catA, catA11-A14 family and catP, responsible for inactivating chloramphenicol [48], are present exclusively in disease-associated subgroups, PM89KC-G-1, PM89KC-G-2 and PM89KC-AC-1 strains, indicating strain-specific resistance to chloramphenicol. The AMR heatmap highlights the heterogeneity in resistance gene profiles among * P. micra* isolates, emphasizing the importance of considering strain-specific factors when making antimicrobial treatment decisions [49]. The distribution of genes like tet(M), ParC and catA suggests varying levels of resistance to specific antibiotics among strains, underscoring the need for tailored therapeutic approaches based on individual resistance profiles.

**Fig. 4.**
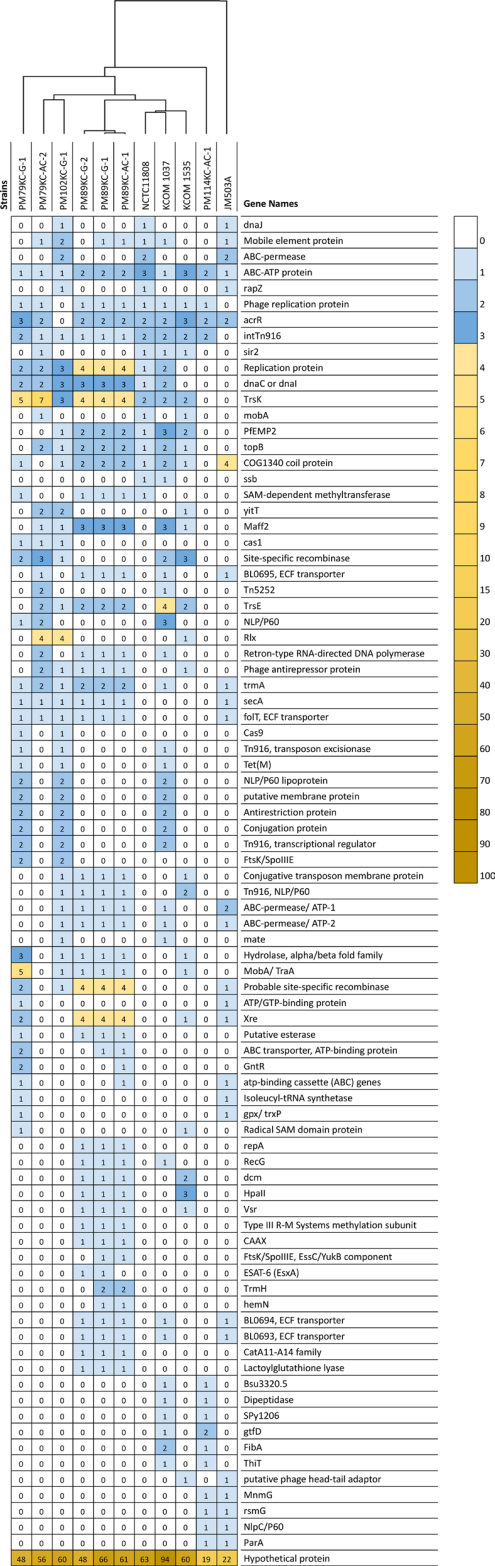
Heatmap of VF gene distribution in 11 *P*. *micra* isolates. A total of 102 accessory VF genes were identified by the PATRIC database in all 11 strains. The copy number, ranging from 0 to 100, was indicated by white to brown. The hierarchical clustering showing the presence of strain-level AMR gene variation in the isolates was presented as a cladogram in the panel along the x-axis.

The RAST subsystems related to antibiotic and toxic compound resistance revealed diverse resistance profiles among the *P. micra* strains, as shown in Fig. 4A. Strain JM503A exhibits a unique adaptation to d-cysteine, suggesting a specialized metabolic adaptation that may impact its survival or resistance in specific environments [50]. Bile hydrolysis, essential for survival in bile-rich environments like the gastrointestinal tract [51], was only observed in strains PM79KC-G-1, KCOM 1037 and PM114KC-AC-1. Resistance to heavy metals like cobalt, zinc and cadmium was present in all strains, indicating a widespread resistance mechanism likely supported by efflux pumps, which also contribute to AMR [52]. These efflux pumps, found in all strains, help bacteria eliminate toxic substances, including antibiotics, hinting at potential multidrug resistance [53]. The presence of copper homeostasis, which is crucial for regulating intracellular copper levels and preventing toxicity [54], suggests additional resistance mechanisms against copper-based treatments. Fluoroquinolone resistance was detected in all strains, possibly due to mutations in DNA gyrase or topoisomerase IV, the main targets of these antibiotics [55]. The presence of the *Streptococcus pneumoniae* vancomycin tolerance locus in multiple strains indicates potential tolerance to vancomycin, posing challenges in treating Gram-positive infections [56]. These findings show the complex and diverse resistance profiles of *P. micra* strains, emphasizing the challenges in antimicrobial therapy due to evolved resistance mechanisms in clinical and environmental settings.

### Virulence factors prediction

A total of 85 accessory VF genes in 11 *P*. *micra* strains were identified using the PATRIC database. A heatmap illustrating the distribution of VF genes and hierarchical clustering shows strain-specific variations in VF gene presence (Fig. 4). Among the analysed strains, genes such as ABC-ATP protein, acrR, phage replication protein and replication protein were widely distributed and found in most strains, including PM79KC-G-1, PM79KC-AC-2, PM102KC-G-1, PM89KC-G-2, PM89KC-G-1 and PM89KC-AC-1. These proteins are crucial for nutrient transport and antibiotic resistance, enhancing adaptability and pathogenic potential [57]. The acrR gene and TetR/AcrR family transcriptional regulators, involved in multidrug resistance and virulence gene expression control, were present in all except in PM102KC-G-1, suggesting unique regulatory mechanisms in these strains [58]. Interestingly, most strains, including NCTC11808, PM79KC-AC-2, PM102KC-G-1, PM89KC-G-2, PM89KC-G-1, PM89KC-AC-1, KCOM 1037 and KCOM 1535, carried the topB gene, vital for DNA replication and repair [59]. Strain JM503A formed a separate cluster from the other strains, likely due to key genomic variations. This limited shared VF count indicates the strain’s distinct genomic profile, potentially leading to reduced virulence or a unique adaptation strategy. Strain-specific genomic features contribute to diverse virulence profiles, underscoring the importance of understanding these variations in the pathogenic potential of *Parvimonas* species.

The accessory virulence gene counts obtained from the IslandViewer database varied from 3 to 51 genes among 11 *P*. *micra* strains (Table S6). Strain-specific variations in VF sharing are evident, with strains PM114KC-AC-1 and PM102KC-G-1 sharing three genes compared with the disease-associated subgroup, strains PM89KC-G-1 and PM89KC-AC-1, which share 51 VFs. The distribution of virulence, disease and defence-related genes identified by the RAST server varied across the 11 *P*. *micra* strains (Fig. 4B). The genes are categorized into subsystem groups based on SEED classification. A notable finding is the presence of invasion and intracellular resistance genes across all strains, highlighting their importance in bacterial pathogenicity [60].

### Mobile genetic elements profile

A detailed breakdown of the number of phages and prophages identified by RAST in 11 *P*. *micra* strains is illustrated as a graph in Fig. S5, categorized according to SEED classification. The presence of phage replication machinery in all strains indicates widespread integration of MGEs, potentially facilitating horizontal gene transfer and contributing to genomic diversity and adaptation in *P. micra* [61]. Phage-related elements, such as replication machinery, can aid in the dissemination of antibiotic resistance or virulence genes among bacterial populations, enhancing their survival and pathogenicity [62]. Distinct strain-specific patterns of MGEs were also observed. Strain NCTC11808 stands out for its possession of phage packaging machinery, a key component involved in assembling and releasing new phage particles. This machinery enables efficient packaging of phage DNA into capsids, crucial for phage propagation within the bacterial host. The unique presence of phage packaging machinery in NCTC11808 suggests a specific evolutionary trajectory or adaptation that may impact its interactions with phages or contribute to its genomic flexibility [63]. In contrast, KCOM 1037 exhibits a more extensive array of phage-associated elements. Alongside phage packaging machinery, this strain harbours phage tail proteins, phage introns and phage capsid proteins, indicating a complex and active phage life cycle. Phage tail proteins facilitate attachment and DNA injection into the bacterial host, while phage introns may regulate gene expression or disrupt host genomic sequences [63,64]. The presence of multiple phage-related proteins in KCOM 1037 suggests a heightened capacity for phage interactions, potentially driving genomic innovation through gene transfer events.

### Carbohydrate-active enzymes

A comparison of CAZymes in 11 *P. micra* strains, showing significant variation within the carbohydrate esterase (CE), glycoside hydrolase (GH) and glycosyltransferase (GT) families, is shown in Table S7. CAZymes are crucial for carbohydrate breakdown and glycoconjugate modification, essential for bacterial metabolism and pathogenesis [65]. The GT119 family is consistently present in all strains with three copies, likely involved in cell wall biosynthesis. GT51, associated with protein glycosylation, is found in all strains with one copy, potentially impacting host–pathogen interactions. GT134 is absent in KCOM 1037 but present in other strains, affecting sugar moiety transfer and pathogenicity. GT2 and GT28 are equally distributed with two copies in all strains, playing roles in polysaccharide and peptidoglycan biosynthesis, respectively [66]. GT4 family is present in all strains, but with a lower count of 1 in NCTC11808, KCOM 1037 and KCOM 1535. GT4 enzymes are involved in energy metabolism, and their reduced presence may suggest a diminished capacity for polysaccharide modification in these strains [67]. GH23 and GH73 families are both evenly distributed across all strains, except for GH23, which is completely absent in NCTC11808 and PM102KC-G-1. GH23 is a family of lysozymes that hydrolyse bacterial cell walls, potentially playing a role in inter-bacterial competition or defence against phage attacks [68]. GH73 enzymes are involved in peptidoglycan degradation and may contribute to remodelling bacterial cell walls during growth or infection [69]. The CE4 family is present in only five strains, with a higher count of 2 in KCOM 1037 and a lower count of 1 in NCTC11808, PM102KC-G-1, KCOM 1535 and JM503A. CE4 enzymes typically deacetylate polysaccharides, which may enhance bacterial survival by modifying host glycan structures. The varying presence of CE4 across strains could indicate different environmental adaptation strategies or metabolic requirements for each strain [70].

### Core and pan-genome analysis

An in-depth pan-genome analysis of 11 *P*. *micra* strains, offering insights into the genetic diversity and evolutionary patterns within the species, is illustrated in Fig. S6. The distribution of gene families across the 11 strains shows the balance between core, accessory and unique genes (Fig. 6A). The core genome represents the set of genes shared by all strains, indicating essential functions for survival, while accessory and unique genes reflect strain-specific adaptations. Fig. 6B shows the sequential addition of each genome to the analysis, revealing the introduction of new gene families. This stepwise process emphasizes the pan-genomic diversity of *P. micra*, indicating an open pangenome [71]. As each strain is included, new gene families are identified, indicating ongoing evolution and the acquisition of new genes, possibly through horizontal gene transfer or adaptation to different environments [72]. The core-pan plot (Fig. 6C) provides a graphical representation of the relationship between total gene families and core gene families. As more genomes are added, the core genome stabilizes, but the pan-genome expands, reflecting the species’ genetic diversity. This trend is typical of bacterial populations that inhabit diverse environments, showcasing the adaptive capabilities of *P. micra*. Protein sequences from core (1,166), accessory (2,944) and unique (440) genes and exclusively absent genes (76 genes) were identified, and their functions in COG and Kyoto Encyclopedia of Genes and Genomes (KEGG) pathways were determined. Exclusively absent genes are those present in the overall pan-genome but completely missing from specific strains. These genes help in identifying genomic signatures that differentiate certain strains within a species [34]. Accessory genes, found in only a few genomes, are mainly related to cellular metabolism, indicating adaptations to specific environments. Unique genes, specific to individual strains, are primarily involved in information processing, suggesting specialized cellular functions (Fig. 6D and 6E). The unique genes of the 11 *P*. *micra* strains were further analysed to uncover genetic features contributing to strain-specific survival and adaptation to different niches.

**Fig. 5.**
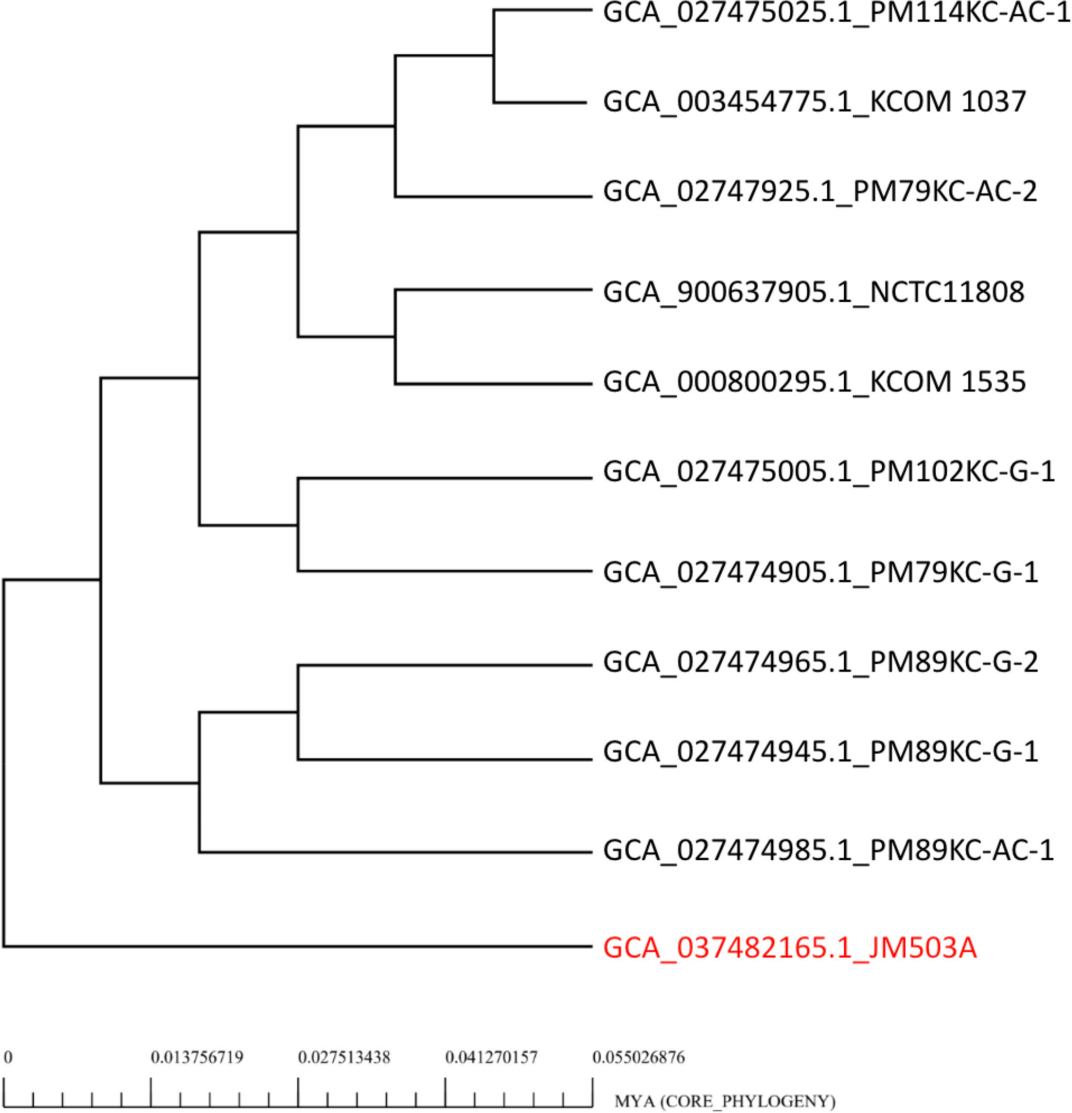
Core genome-based phylogenetic tree of 11 *P*. *micra* strains constructed using the BPGA pipeline. The tree illustrates evolutionary relationships inferred from core gene sequences. The time scale in million years ago (MYA) was generated by BPGA using molecular clock assumptions applied to the aligned core genome data. Bootstrap values are indicated at major nodes. The strain JM503A, forming a separate clade was highlighted in red colour.

The core phylogeny based on the pan-genome of *P. micra* strains is depicted in Fig. 5. The tree is divided into three clusters. The ten strains, except JM503A, are grouped into two clusters. These strains are closely related, suggesting common evolutionary histories or ecological niches. Strain JM503A, isolated from the oral cavity, forms a distinct lineage highlighted in the red box. This strain has evolved significantly, possibly due to its adaptation to the oral environment.

### COG category distribution

**Fig. 6.**
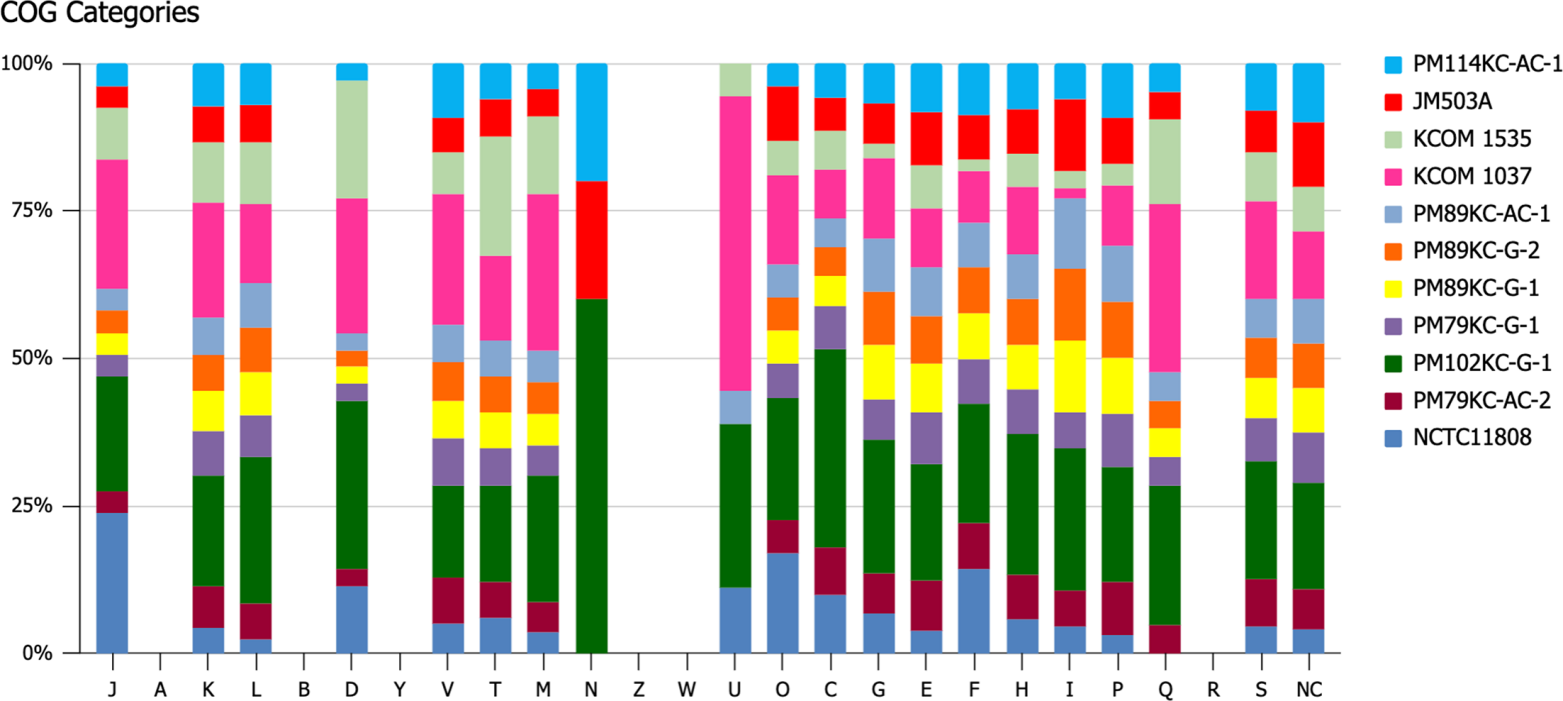
Distribution of COG functional categories. The COG function classifications of consensus sequences were presented graphically. The categories of the COG are shown on the horizontal axis, and genomes are plotted on the vertical axis with different colour codes.

The analysis of COG functional categories among *P. micra* strains revealed significant variation in structural and functional genes related to metabolism and regulation (Fig. 6). PM102KC-G-1 exhibited the highest number of COGs, indicating a broader functional capacity, while NCTC11808 had the least, suggesting reduced functional diversity. Notably, the most prominent COG categories included inorganic ion transport and metabolism (P), transcription (K) and aa transport and metabolism (E) with 253, 209 and 208 genes, respectively, highlighting the strains’ metabolic and regulatory potential. Additionally, replication, recombination and repair (L), which contains 201 genes, plays a critical role in maintaining genomic stability, particularly in strains KCOM 1037 and PM102KC-G-1. The numbers in cell motility (N) had minimal representation, with only five genes. The COG functional categories of translation, ribosomal structure and biogenesis (J); transcription (K); and replication, recombination and repair (L) showed between 30 and 50 differential genes, indicating robust growth and genomic repair mechanisms, while the numbers in other groups are <30. In contrast, the intracellular trafficking and secretion and vesicular transport (U) category exhibited variability, with KCOM 1037 and PM102KC-G-1 showing higher gene counts, while strains PM89KC-AC-1 and KCOM 1535 had lower counts, reflecting differences in cellular transport capabilities. Among all strains, COG categories associated with inorganic ion transport and metabolism, transcription, aa transport and metabolism, and replication, recombination and repair were frequently mapped, indicating their significance in the survival and adaptability of *P. micra* strains. The distribution of COG categories highlights the functional diversity of these strains, with potential implications for their pathogenicity, survival strategies and resistance mechanisms [73].

### Taxonomic assessment of strain JM503A

To further validate the findings on the distinctiveness of strain JM503A, we constructed a phylogenetic tree with 44 genomes from taxonomically validated *Parvimonas* spp. (*P. micra* and *Parvimonas parva*) and 81 genomes of the *Parvimonas* genus including the draft genomes with different levels of completeness. Core-genome phylogeny of 44 genomes indicated that strain JM503A, highlighted in red colour, occurred in a separate phylogenetic lineage, closest to but in separate phylogenetic lineages from * P. parva*, highlighted in blue colour, and the strains having uncertain taxonomy, highlighted in brown colour (Fig. S7). The expanded core-genome analyses using 81 *Parvimonas* genomes with JM503A are indicated in red colour and inconclusive strains in blue colour (Fig. S8), plus ANI and DDH analyses (Fig. S9A, B) (only with representative genomes from each phylogenetic cluster), all of which supported the observation (above) that JM503A is uniquely placed.

We also conducted genome-based taxonomic predictions using the GermAI (EzBioCloud) server [74], which predicted a potential affiliation with the genus *Peptostreptococcus* (Fig. S10A, B). We conducted 16S rRNA analyses using 26 *Peptostreptococcus* reference species from the LPSN database, along with 11 *P*. *micra* strains. Strain JM503A, highlighted in red colour, shows some genetic proximity to *Peptostreptococcus*, highlighted in blue colour, but did not cluster in any of the recognized species of *Peptostreptococcus* (Fig. S11). Further, the ANI and DDH comparison using only two available complete genomes of *Peptostreptococcus* supports this analysis (Table S12A, B). Further, a 16S rRNA-based phylogenetic tree was constructed using 11 *P*. *micra* genomes and two *Peptostreptococcus* genomes. Strain JM503A, in red colour, formed a distinct branch adjacent to the *Peptostreptococcus* clade but remained genetically distant from it, indicating that it does not belong to the genus *Peptostreptococcus* and may represent a novel taxonomic entity (Fig. S13).

While genome-based taxonomic predictions and 16S rRNA analyses initially provided indications of a genus-level affiliation with *Peptostreptococcus*, the ANI and dDDH values did not support this classification. To reassess this classification, we conducted a phylogenomic analysis with genomes of 48 *Peptostreptococcus* strains*,* 2 *P*. *parva* strains and 11 *P*. *micra* strains, including JM503A (Fig. S14). Phylogenomic analyses revealed that JM503A formed a distinct clade with the *Parvimonas* genus, which was separated from the *Peptostreptococcus* genomes, providing strong support for considering *P. micra* strain JM503A as a new species in the genus *Parvimonas*.

### Recommendation to reclassify *P. micra* strain JM503A as a novel species

Strain JM503A, identified as a novel species of the genus *Parvimonas* by phylogenetic and comparative genome analysis, exhibited several key characteristics, detailed in Table 3. The GC content of strain JM503A was measured at 27.9% which is in line with the typical range for anaerobic bacteria, reflecting genome stability and adaptation to its niche [75]. Further, it may reflect distinct genomic adaptations and support its divergence from typical *P. micra* strains, consistent with its proposed reclassification as a novel species within the genus. The genome size of 1,549,037 bp demonstrates a relatively compact genome size that aligns with other strains within the *P. micra* group. The 16S rRNA gene sequence similarity between JM503A and other known *P. micra* strains was calculated to be 98.28%. This percentage suggests close phylogenetic relatedness, but is below the 98.7-99% threshold typically used for bacterial species demarcation [76], supporting the notion that JM503A could be a novel species. Whole genome similarity values, based on the Gegenees heatmap, revealed a range of 54–61% similarity with other strains, also suggesting that JM503A may represent a distinct lineage with moderate genomic overlap with existing *P. micra* species. The pairwise ANI values of JM503A compared with other *P. micra* strains ranging from 91.32% to 91.7%, further affirm the distinctiveness of this strain, as ANI values below 95% generally suggest species-level divergence. Lastly, DDH values ranging from 43.00% to 44.00%, which are well below the 70% cutoff traditionally used to delineate bacterial species, further confirm the strain’s novelty. Whole-genome-based phylogeny and comparative genome analysis have been incorporated in a polyphasic approach for describing novel bacterial species [42]. Hence, based on the phylogenetic and comparative genomic characteristics of JM503A, we recommend this strain to be described as a novel species within the *Parvimonas* genus.

**Table 3.** Summary of key characteristic features of the strain JM503A of *P. micra*.The table presents a comprehensive overview of the key characteristics associated with strain JM503A, along with the various tools and methodologies employed to identify the features.

Characteristic	Strain JM503A	Other strain	Tool
GC content	27.9	28.5–29.1	RAST server
Genome size	15,49,037 bp	–	RAST server
16S %Similarity	98.28%	98.7–99.0%	blastn tool
Whole-genome similarity	54–61%	Above 75%	Gegenees software
ANI	91%	Above 95%	EzBioCloud ANI tool
dDDH	43.00–44.00%	Above 70%	GGDC database

## Supplementary material

10.1099/mgen.0.001511 Uncited Supplementary Material 1.

## Abbreviations

AMR: antimicrobial resistance
ANI: average nucleotide identity
blastn: Basic Local Alignment Search Tool (nucleotide)
BPGA: Bacterial Pan Genome Analysis
CAZyme: carbohydrate-active enzyme
CDS: coding sequence
CE: carbohydrate esterase
COG: clusters of orthologous gene
CRC: colorectal cancer
dDDH: digital DNA–DNA hybridization
DDH: DNA–DNA hybridization
GCF: gingival crevicular fluid
Gegenees: genome evolutionary genealogy employing ensemble statistics
GGDC: Genome-to-Genome Distance Calculator
GH: glycoside hydrolase
GT: glycosyltransferase
MEGA: molecular evolutionary genetics analysis
MGE: mobile genetic element
MUSCLE: MUltiple Sequence Comparison by Log-Expectation
NCBI: National Center for Biotechnology Information
PATRIC: Pathosystems Resource Integration Center
RAST: Rapid Annotation using Subsystem Technology
SEED: System for Effective and Efficient Discovery
VF: virulence factor
