# Supplementary material for: Comparative genome analysis of human pathogen Parvimonas micra revealed strain JM503A as potential novel species in the genus Parvimonas and high intra-species functional diversity
Source: Microb Genom. 2025 Sep 22;11(9):001511. doi: 10.1099/mgen.0.001511 (PMC12476148; doi:10.1099/mgen.0.001511)
Supplement: Uncited Supplementary Material 1. [file mgen-11-01511-s001.pdf]

Supplementary Figure 1. Comparative analysis of whole genome sequence data performed using the Gegenees software. The heat-plot was based on a fragmented alignment using BLASTN with settings 50/25. The matrix displays percentage identity values, with color intensity indicating similarity (green: high; yellow: moderate; orange: low, strain JM503A). The upper-right and lower-left triangles represent results obtained using different fragment sizes, allowing comparative resolution of genomic similarities.

Organism	1	2	3	4	5	6	7	8	9	10	11
1: GCA_000800295.1_KCOM 1535	100	83	81	85	86	82	79	82	82	82	58
2: GCA_900637905.1_NCTC11808	81	100	80	80	82	80	78	80	81	81	56
3: GCA_003454775.1_KCOM 1037	80	82	100	82	83	80	79	80	82	82	57
4: GCA_027474905.1_PM79KC-G-1	83	81	80	100	86	80	77	80	82	82	57
5: GCA_027475005.1_PM102KC-G-1	82	80	79	83	100	79	75	79	80	80	55
6: GCA_027474925.1_PM79KC-AC-2	77	77	76	77	78	100	75	77	78	78	54
7: GCA_027475025.1_PM114KC-AC-1	84	86	86	85	85	86	100	84	85	85	60
8: GCA_027474985.1_PM89KC-AC-1	81	82	80	81	82	81	77	100	96	96	56
9: GCA_027474945.1_PM89KC-G-1	81	82	81	82	83	81	77	95	100	100	57
10: GCA_027474965.1_PM89KC-G-2	81	82	81	82	83	81	77	95	100	100	56
11: GCA_037482165.1_JM503A	60	61	61	61	61	61	59	60	61	61	100

Supplementary Figure 2. A heatplot was generated to visualize the average nucleotide identity (ANI) values among the 11 strains, calculated using the OrthoANIu algorithm implemented in the EzBiocloud ANI calculator, which utilizes USEARCH for sequence comparison. Color gradient indicates degree of similarity: red (≥99%, identical or nearly identical), yellow (90–98%, moderate identity), and green, strain JM503A (<92%, low identity).

ANI	NCTC11808	PM79KC-AC-2	PM102KC-G1	PM79KC-G-1	PM89KC-G-1	PM89KC-G-2	PM89KC-AC-1	KCOM 1037	KCOM 1535	JM503A	PM114KC-AC-1
NCTC11808	100	96.94	97.27	97.4	97.26	97.25	97.2	97.57	97.38	91.71	97.35
PM79KC-AC-2	96.94	100	96.7	96.89	96.96	97.02	97.06	96.77	96.8	91.42	97.45
PM102KC-G-1	97.27	96.7	100	97.44	97.18	97.2	97.1	97.25	97.44	91.59	97.19
PM79KC-G-1	97.4	96.89	97.44	100	97.48	97.39	97.34	97.21	97.34	91.45	97.33
PM89KC-G-1	97.26	96.96	97.18	97.48	100	99.95	99.23	97.27	97.23	91.4	97.41
PM89KC-G-2	97.25	97.02	97.2	97.39	99.95	100	99.25	97.33	97.25	91.42	97.43
PM89KC-AC-1	97.2	97.06	97.1	97.34	99.23	99.25	100	97.24	97.41	91.5	97.61
KCOM 1037	97.57	96.77	97.25	97.21	97.27	97.33	97.24	100	97.37	91.15	97.15
KCOM 1535	97.38	96.8	97.44	97.34	97.23	97.25	97.41	97.37	100	91.32	97.29
JM503A	91.71	91.42	91.59	91.45	91.4	91.42	91.5	91.15	91.32	100	91.5
PM114KC-AC-1	97.35	97.45	97.19	97.33	97.41	97.43	97.61	97.15	97.29	91.5	100

Supplementary Figure 3. Pairwise comparison of digital DDH values among *P. micra* genomes sequenced to date. The digital DDH values were calculated using the DSMZ digital DDH calculation tool with the option for BLAST. Shaded in yellow and red are DDH values >70 % corresponding to the same species of *P. micra,* and shaded in green (strain JM503A) are DDH values of <70 % with each other corresponding to different species.

DDH Value	NCTC11808	PM79KC-AC-2	PM102KC-G1	PM79KC-G-1	PM89KC-G-1	PM89KC-G-2	PM89KC-AC-1	KCOM 1037	KCOM 1535	JM503A	PM114KC-AC-1
**NCTC11808**	100.00%	70.90%	73.50%	74.50%	74.40%	74.50%	74.00%	75.20%	74.90%	44.00%	74.70%
**PM79KC-AC-2**	70.90%	100.00%	69.50%	71.50%	72.50%	72.50%	72.40%	69.70%	70.30%	43.30%	76.90%
**PM102KC-G-1**	73.50%	69.50%	100.00%	75.80%	73.60%	73.60%	73.40%	72.70%	75.90%	43.00%	74.90%
**PM79KC-G-1**	74.50%	71.50%	75.80%	100.00%	74.60%	74.70%	73.70%	72.50%	76.90%	43.40%	75.50%
**PM89KC-G-1**	74.40%	72.50%	73.60%	74.60%	100.00%	100.00%	93.30%	73.20%	74.50%	43.40%	76.00%
**PM89KC-G-2**	74.50%	72.50%	73.60%	74.70%	100.00%	100.00%	93.30%	73.30%	74.60%	43.40%	76.10%
**PM89KC-AC-1**	74.00%	72.40%	73.40%	73.70%	93.30%	93.30%	100.00%	72.80%	74.20%	43.60%	76.00%
**KCOM 1037**	75.20%	69.70%	72.70%	72.50%	73.20%	73.30%	72.80%	100.00 %	74.40%	43.30%	75.10%
**KCOM 1535**	74.90%	70.30%	75.90%	76.90%	74.50%	74.60%	74.20%	74.40%	100.00%	43.60%	74.30%
**JM503A**	44.00%	43.30%	43.00%	43.40%	43.40%	43.40%	43.60%	43.30%	43.60%	100.00%	43.40%
**PM114KC-AC-1**	74.70%	76.90%	74.90%	75.50%	76.00%	76.10%	76.00%	75.10%	74.30%	43.40%	100.00%

Supplementary Figure 4A. The number of resistance to antibiotics and toxic compounds annotated by RAST for each subsystem group according to the SEED classification, was determined for 11 strains of *P. micra.*

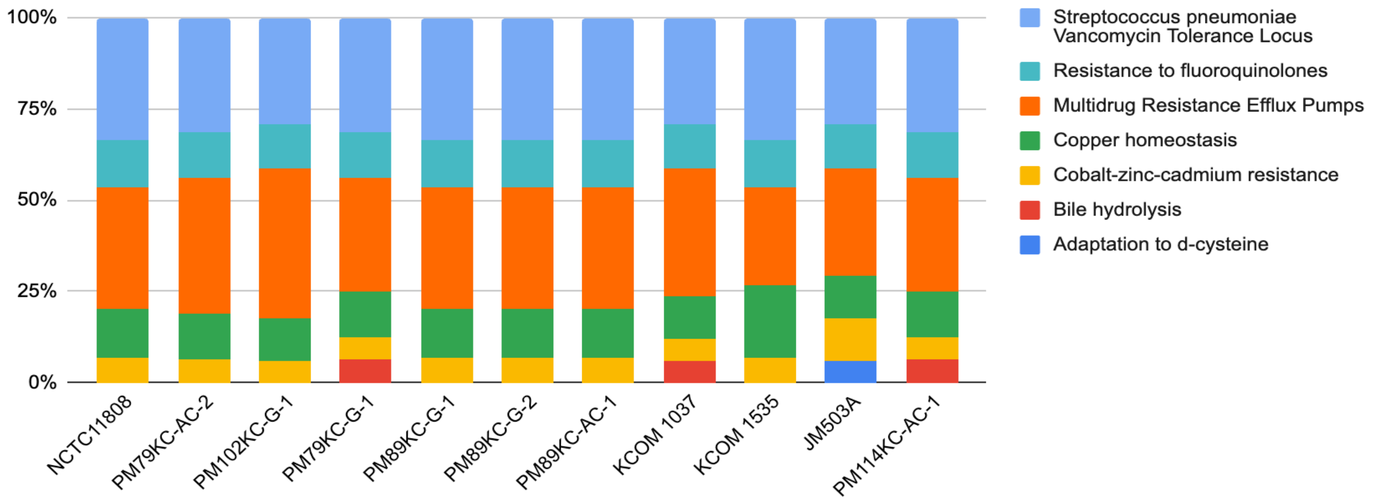

Supplementary Figure 4B. The number of virulence, disease, and defense genes annotated by RAST for each subsystem group according to the SEED classification was determined for 11 strains of *P. micra.*

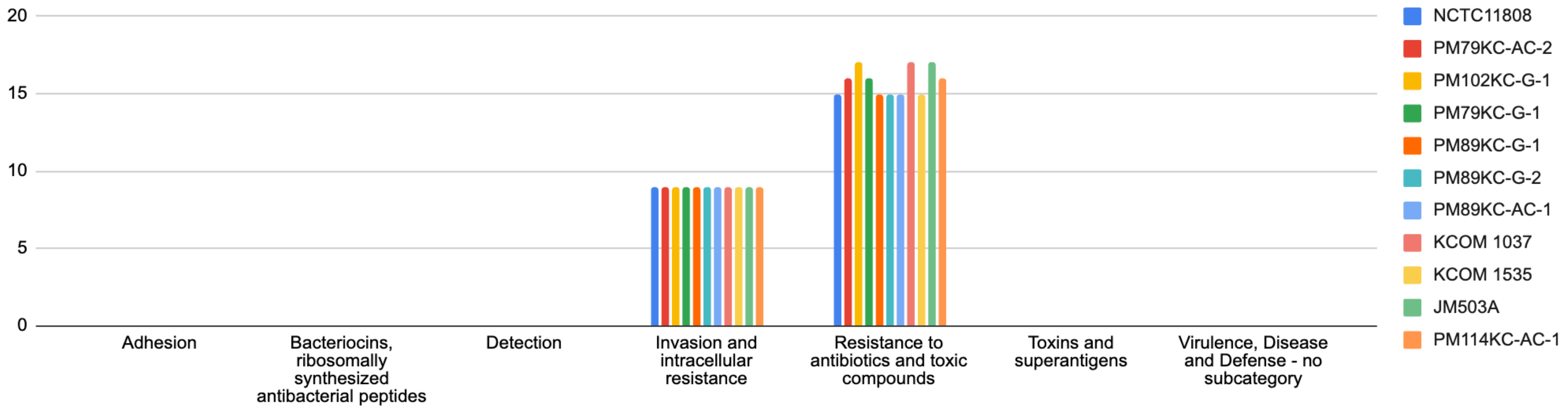

Supplementary Figure 5. The number of phages and prophages annotated by RAST for each subsystem group according to the SEED classification was determined for 11 strains of *P. micra*

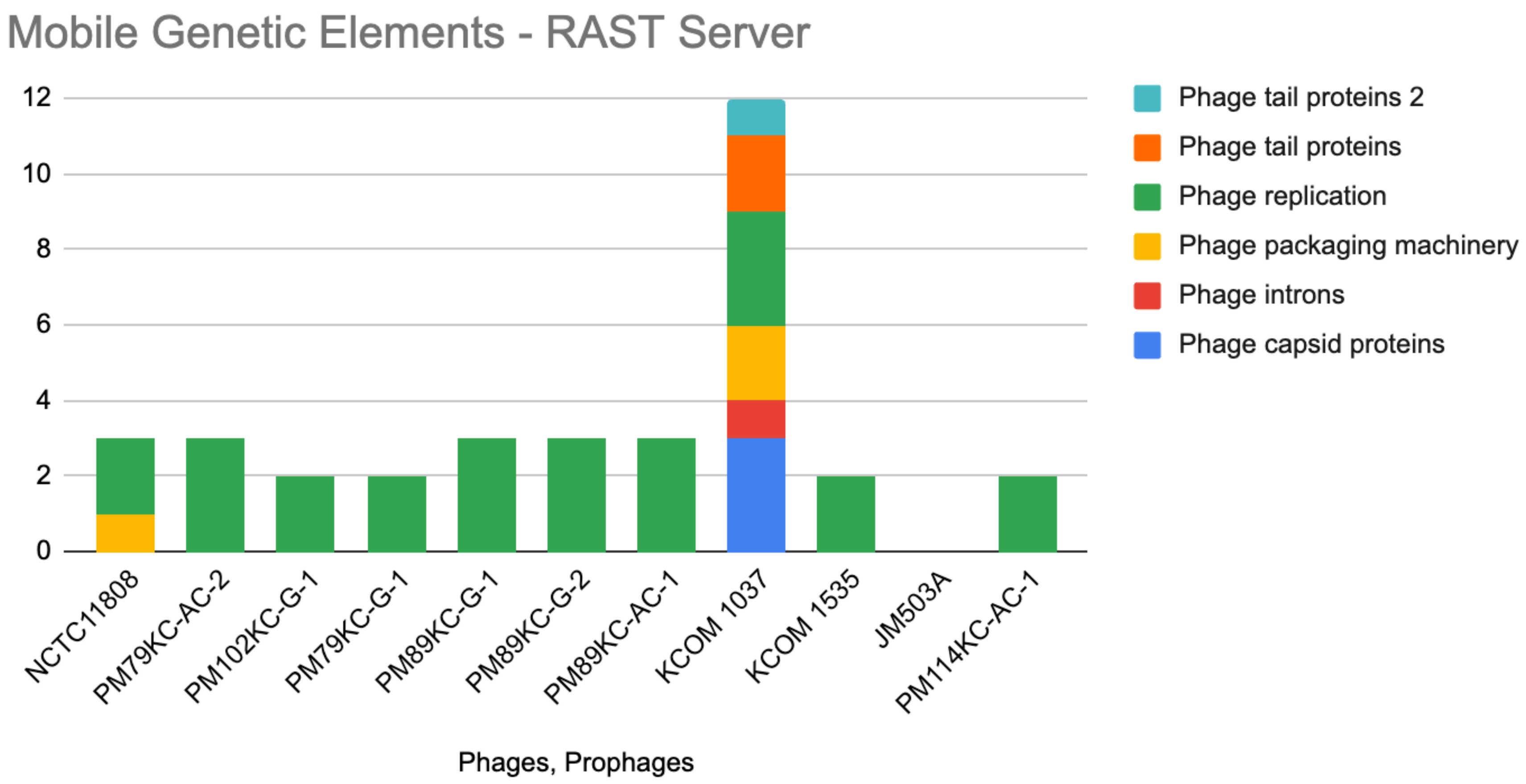

Supplementary Figure 6. Overview of the results generated by BPGA using 11 strains of *P. micra*. (A) The gene family frequency spectrum. (B) New gene family distribution after sequential addition of each genome to the analysis. (C) The core-pan plot, genome profile trends obtained using total gene and core gene families. (D) COG distribution of core, accessory and unique genes. (E) KEGG distribution of core, accessory and unique genes.

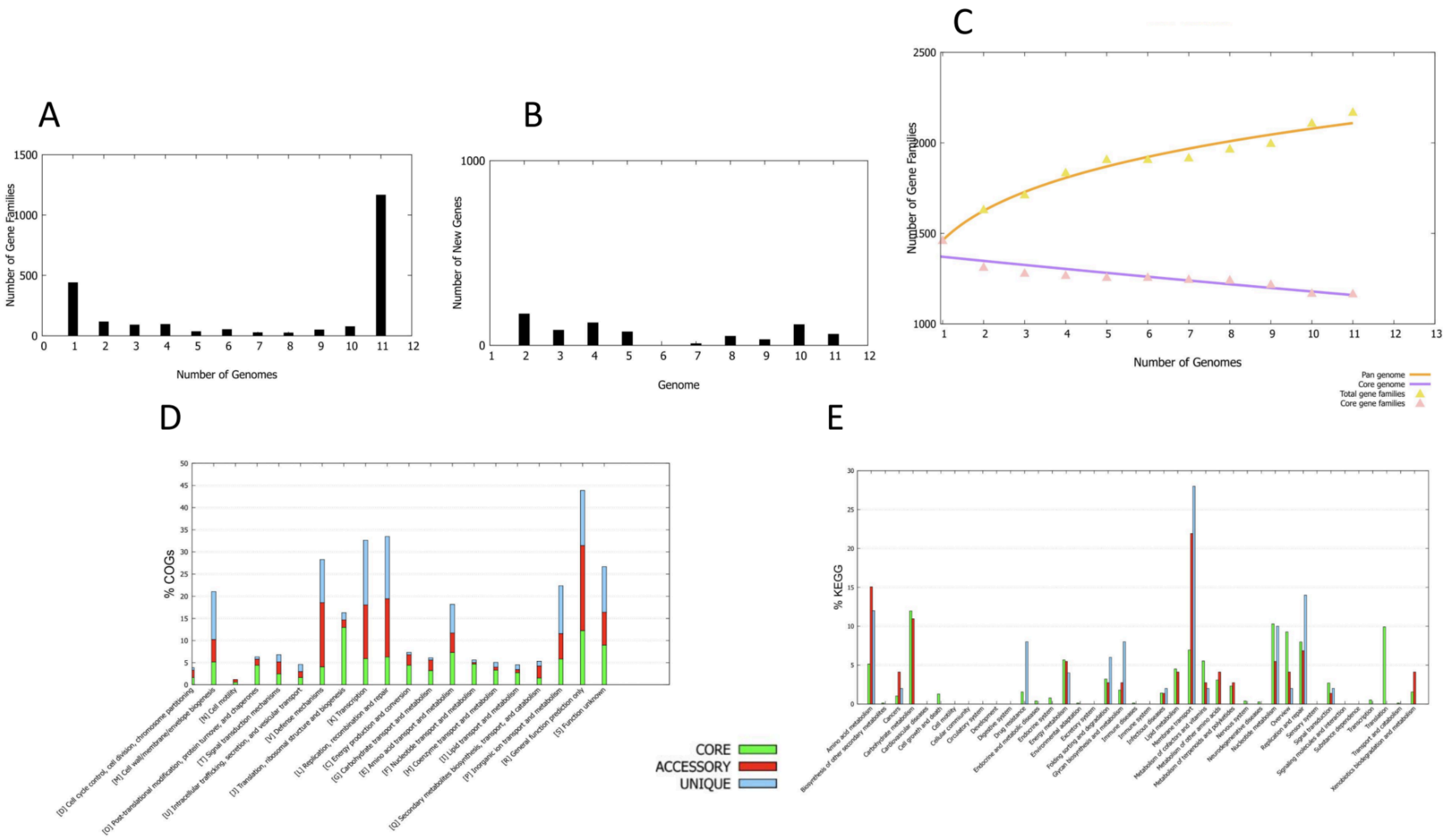

Supplementary Figure 7. Core-genome phylogenetic tree of 44 *Parvimonas* genomes including *P. micra*, *P. parva*, and taxonomically inconclusive strains. Strain JM503A (highlighted in red) forms a distinct lineage, separate from both *P. parva* (highlighted in blue) and unclassified strains (highlighted in brown), suggesting a potentially novel taxonomic position.

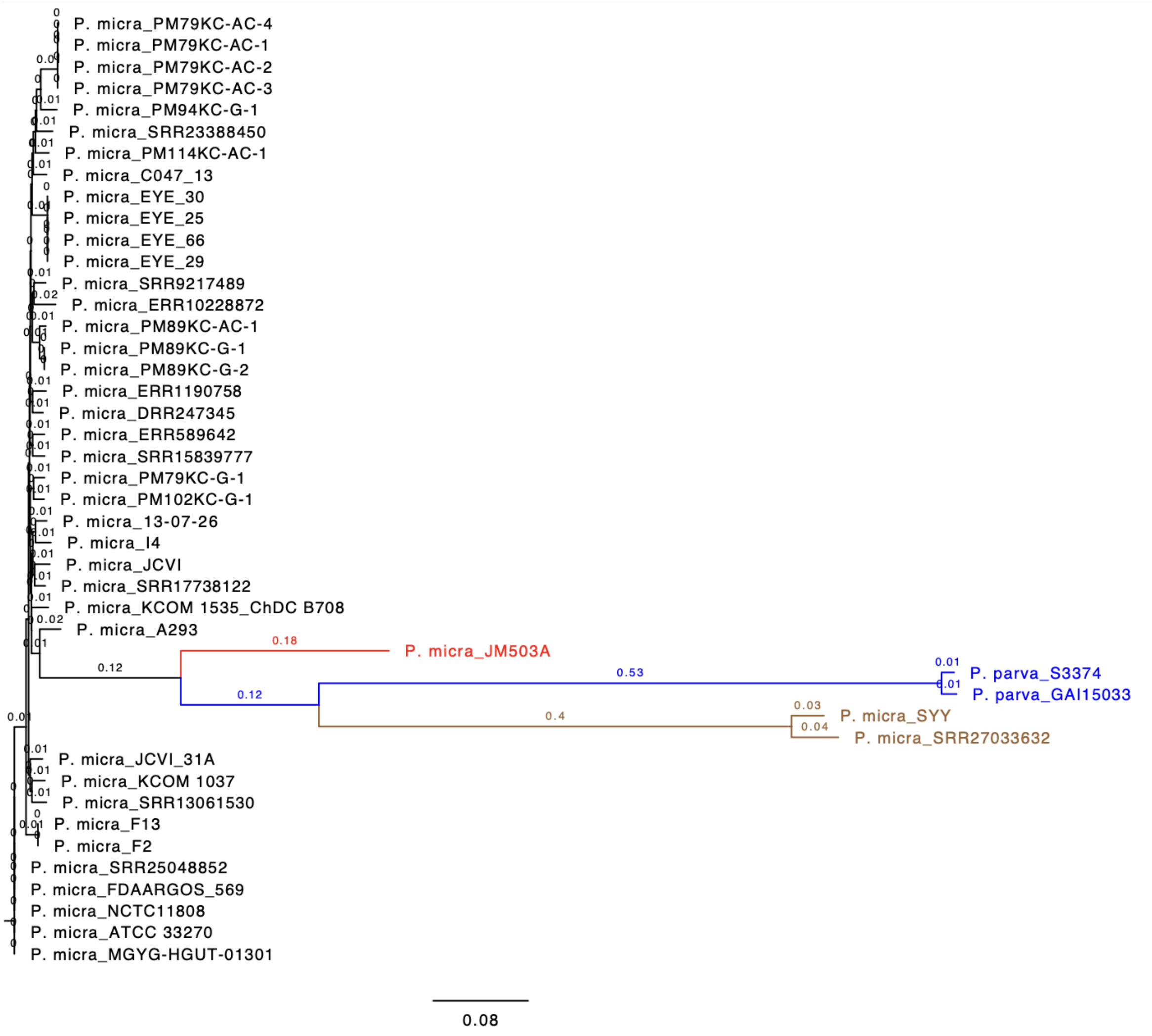

Supplementary Figure 8. Core-genome phylogenetic tree of 81 *Parvimonas* genomes, including strain JM503A (highlighted in red) and taxonomically inconclusive strains (highlighted in blue).

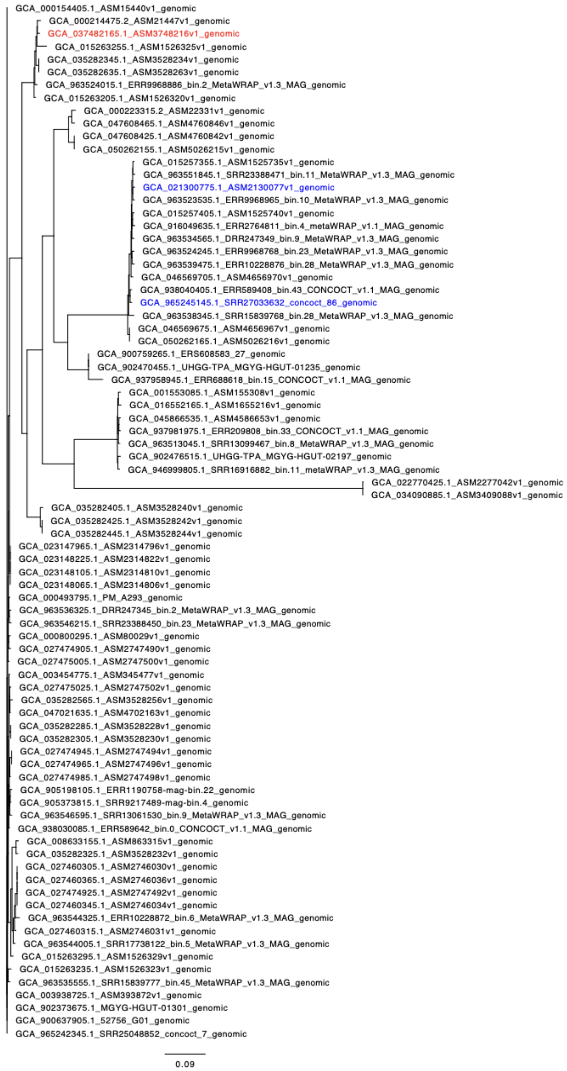

Supplementary Figure 9A. Heatmap of Average Nucleotide Identity (ANI) values among representative *Parvimonas* genomes, including JM503A (highlighted in bold). Color gradient indicates degree of similarity: red (≥99%, identical or nearly identical), orange/yellow (90–98%, moderate identity), and green (<90%, low identity).

ANI		GCA_016552165.1	GCA_045866535.1	GCA_021300775.1	GCA_965245145.1	**GCA_037482165.1**	GCA_027460365.1	GCA_027474925.1	GCA_027460305.1
GCA_016552165.1	S3374	100	97.66	84.32	84.64	85.76	85.64	85.63	85.63
GCA_045866535.1	GAI15033	98.1	100	84.66	84.69	85.95	86.12	86.13	86.14
GCA_021300775.1	SYY	84.45	84.53	100	96.41	86.54	86.42	86.43	86.41
GCA_965245145.1	SRR27033632_concoct_86	84.8	84.88	96.76	100	86.7	86.64	86.64	86.64
**GCA_037482165.1**	**JM503A**	85.85	85.77	86.67	86.47	100	91.48	91.49	91.48
GCA_027460365.1	PM79KC-AC-1	85.63	85.96	86.34	86.67	91.48	100	100	99.96
GCA_027474925.1	PM79KC-AC-2	85.53	85.85	86.36	86.35	91.37	99.9	100	99.88
GCA_027460305.1	PM79KC-AC-4	85.57	85.91	86.42	86.51	91.61	99.99	100	100

Supplementary Figure 9B. Heatmap of Digital DNA - DNA Hybridization (DDH) values among representative *Parvimonas* genomes, including JM503A (highlighted in bold). Color gradient indicates degree of similarity: red (≥99%, identical or nearly identical), orange (70-90%, moderate identity), and yellow/green (<70%, low identity).

DDH		GCA_016552165.1	GCA_045866535.1	GCA_021300775.1	GCA_965245145.1	**GCA_037482165.1**	GCA_027460365.1	GCA_027474925.1	GCA_027460305.1
GCA_016552165.1	S3374	100	83.8	27.6	29.7	30.2	30	30.1	30.1
GCA_045866535.1	GAI15033	83.8	100	27.6	29.6	29.9	30.2	30.3	30.3
GCA_021300775.1	SYY	27.6	27.6	100	72.1	30.8	30.6	30.7	30.6
GCA_965245145.1	SRR27033632_concoct_86	29.7	29.6	72.1	100	32.8	32.6	32.6	32.7
**GCA_037482165.1**	**JM503A**	30.2	29.9	30.8	32.8	100	43.3	43.3	43.6
GCA_027460365.1	PM79KC-AC-1	30	30.2	30.6	32.6	43.3	100	99.8	99.9
GCA_027474925.1	PM79KC-AC-2	30.1	30.3	30.7	32.6	43.3	99.8	100	99.8
GCA_027460305.1	PM79KC-AC-4	30.1	30.3	30.6	32.7	43.6	99.9	99.8	100

Supplementary Figure 10A. Genome-based taxonomic prediction of JM503A strain using GermAI (EzBioCloud) server. The analysis indicates a probable taxonomic affiliation with the genus *Peptostreptococcus*, based on whole-genome similarity metrics.

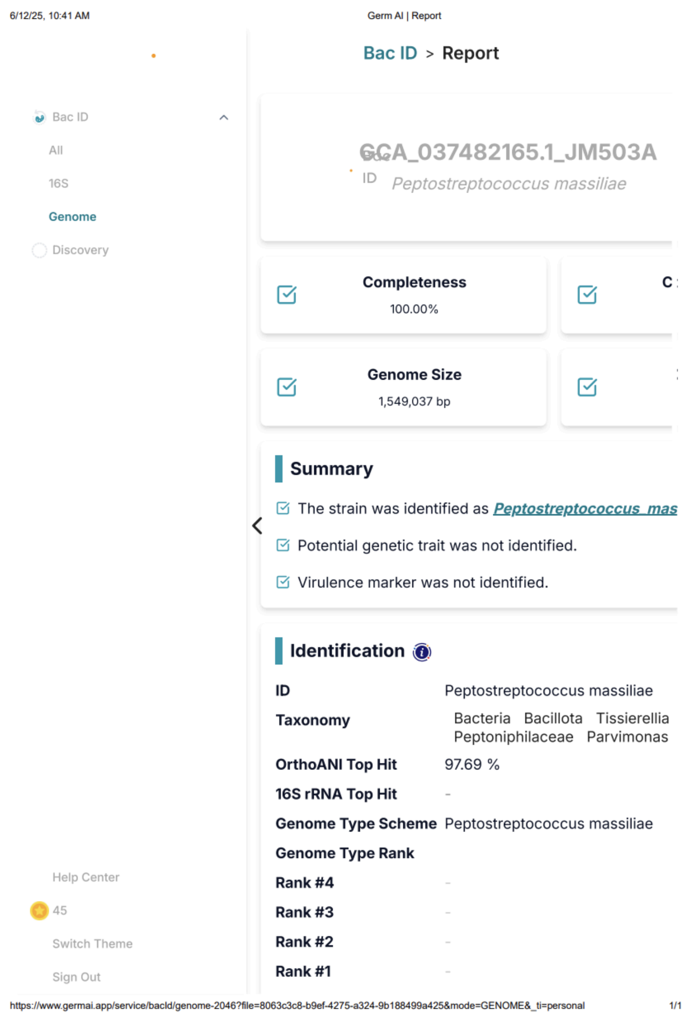

Supplementary Figure 10B. Visual representation of GermAI's genus-level prediction output showing the taxonomic placement of the isolate JM503A within the *Peptostreptococcus* genus, along with a similarity score of 99.64%.

☐	Tasks	Name	Top-hit taxon	Top-hit strain	Similarity (%)	Top-hit taxonomy	Completeness (%)
☐		P. micra PM114KC-AC-1	Parvimonas micra	ATCC 33270	100.00	Bacteria;Bacillota;Tissierellia;Tissierellales;Peptoniphilaceae;Parvimonas	100.0
☐		P. micra JM503A	Peptostreptococcus massiliensis	2002-69396	99.64	Bacteria;Bacillota;Tissierellia;Tissierellales;Peptoniphilaceae;Parvimonas	100.0
☐		P. micra KCOM 1535	Parvimonas micra	ATCC 33270	100.00	Bacteria;Bacillota;Tissierellia;Tissierellales;Peptoniphilaceae;Parvimonas	100.0
☐		P. micra KCOM 1037	Parvimonas micra	ATCC 33270	100.00	Bacteria;Bacillota;Tissierellia;Tissierellales;Peptoniphilaceae;Parvimonas	100.0
☐		P. micra PM89KC-AC-1	Parvimonas micra	ATCC 33270	99.79	Bacteria;Bacillota;Tissierellia;Tissierellales;Peptoniphilaceae;Parvimonas	100.0
☐		P. micra PM89KC-G-2	Parvimonas micra	ATCC 33270	99.79	Bacteria;Bacillota;Tissierellia;Tissierellales;Peptoniphilaceae;Parvimonas	100.0
☐		P. micra PM89KC-G-1	Parvimonas micra	ATCC 33270	99.79	Bacteria;Bacillota;Tissierellia;Tissierellales;Peptoniphilaceae;Parvimonas	100.0
☐		P. micra PM79KC-G-1	Parvimonas micra	ATCC 33270	100.00	Bacteria;Bacillota;Tissierellia;Tissierellales;Peptoniphilaceae;Parvimonas	100.0
☐		P. micra PM102KC-G-1	Parvimonas micra	ATCC 33270	100.00	Bacteria;Bacillota;Tissierellia;Tissierellales;Peptoniphilaceae;Parvimonas	100.0
☐		P. micra PM79KC-AC-2	Parvimonas micra	ATCC 33270	100.00	Bacteria;Bacillota;Tissierellia;Tissierellales;Peptoniphilaceae;Parvimonas	100.0
☐		P. micra NCTC11808	Parvimonas micra	ATCC 33270	100.00	Bacteria;Bacillota;Tissierellia;Tissierellales;Peptoniphilaceae;Parvimonas	100.0

Supplementary Figure 11. 16S rRNA gene-based phylogenetic tree constructed using 26 *Peptostreptococcus* reference species from the LPSN database and 11 *Parvimonas micra* strains. The isolate JM503A (highlighted in red color) shows genetic proximity to the *Peptostreptococcus* clade (highlighted in blue color).

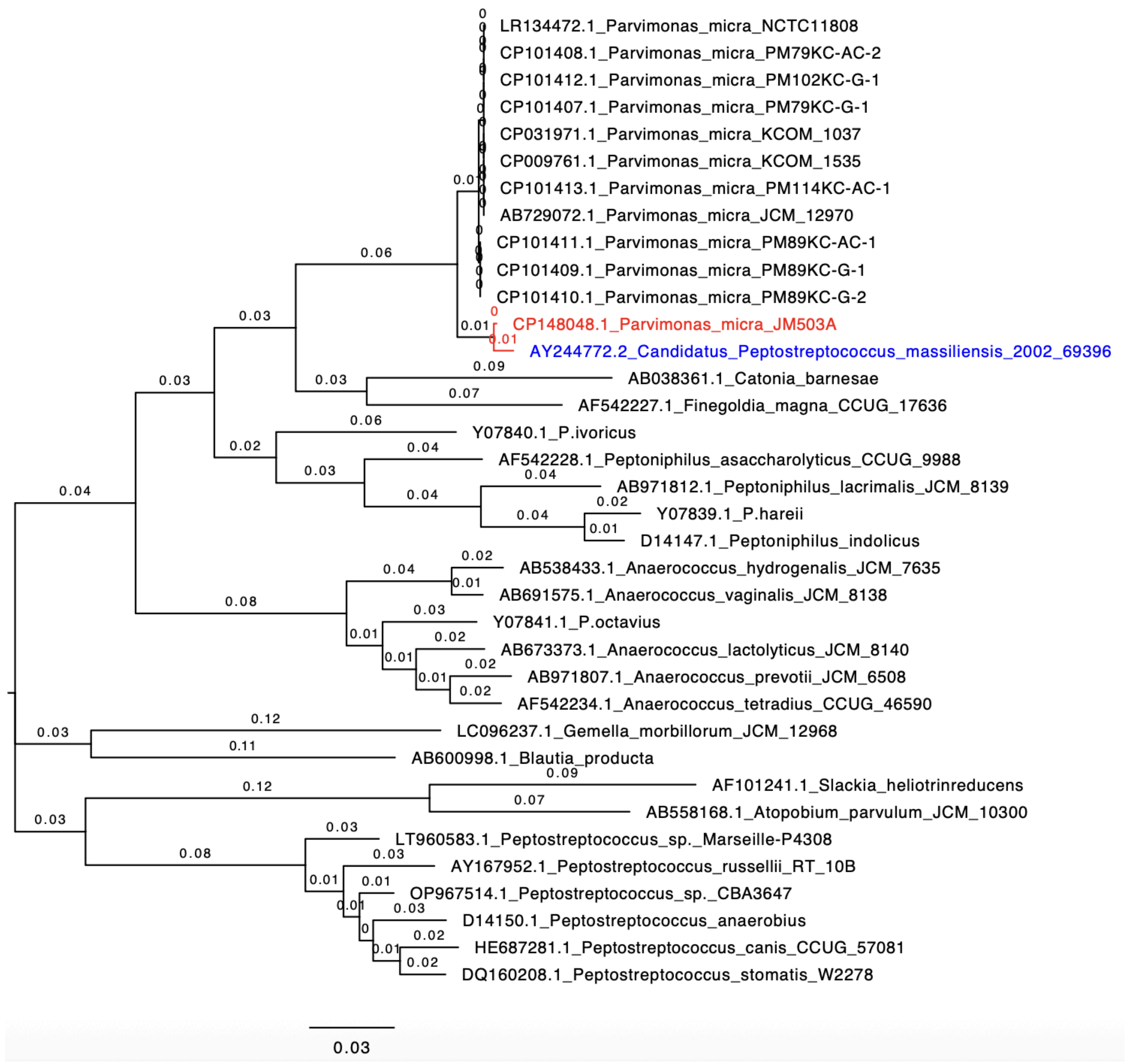

Supplementary Figure 12A. Heatmap of Average Nucleotide Identity (ANI) values among *P. micra* JM503A (highlighted in bold) and 2 complete genomes of *Peptostreptococcus* genus. Color gradient indicates degree of similarity: red (≥99%, identical or nearly identical), orange/yellow (90–98%, moderate identity), and green (<90%, low identity).

**ANI**	**GCA_037482165.1**	GCA_027125355.1	GCA_050780165.1
**GCA_037482165.1_JM503A**	100	65.8	66.1
GCA_027125355.1	66.04	100	72.92
GCA_050780165.1	65.81	72.34	100

Supplementary Figure 12B. Heatmap of Digital DNA - DNA Hybridization (DDH) values among *P. micra* JM503A (highlighted in bold) and 2 complete genomes of *Peptostreptococcus* genus. Color gradient indicates degree of similarity: red (≥99%, identical or nearly identical), orange (70-90%, moderate identity), and yellow/green (<70%, low identity).

**DDH**	**GCA_037482165.1**	GCA_027125355.1	GCA_050780165.1
**GCA_037482165.1_JM503A**	100	21.60%	26.30%
GCA_027125355.1	21.60%	100	24.30%
GCA_050780165.1	26.30%	24.30%	100

Supplementary Figure 13. 16S rRNA gene-based phylogenetic tree constructed using 11 *Parvimonas micra* genomes and 2 *Peptostreptococcus* genomes. The strain JM503A (highlighted in red color) formed a distinct branch more closely related to the *Peptostreptococcus* clade (highlighted in blue color) rather than clustering within the *P. micra* group, suggesting potential misclassification or a novel taxonomic position.

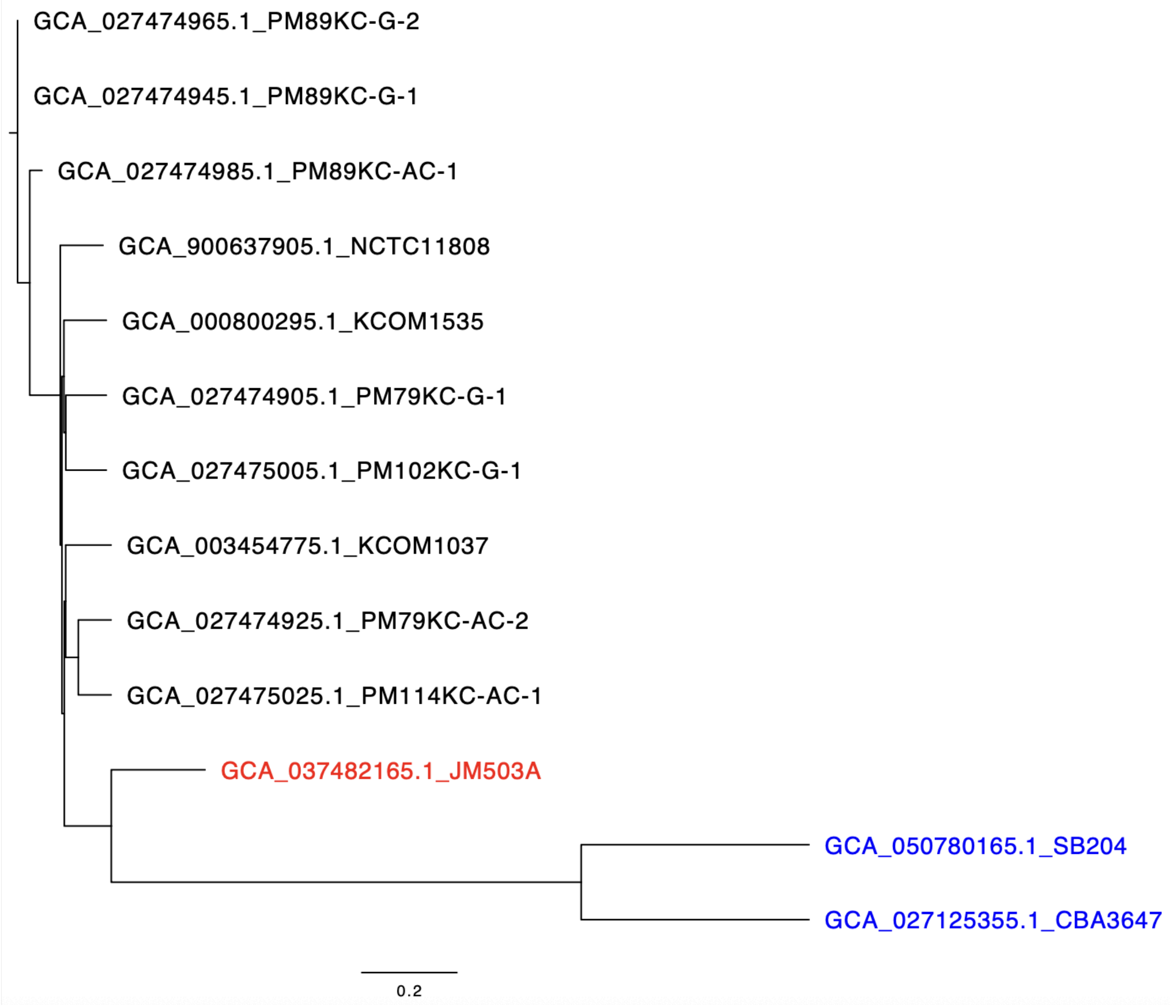

Supplementary Figure 14. Phylogenomic placement of strain JM503A among *Parvimonas* and *Peptostreptococcus* species. Whole-genome phylogenetic tree showing JM503A (red) clustering with *Parvimonas* genomes (blue, Phylotype C), and distinct from *Peptostreptococcus* genomes (Phylotypes A, B, and D). The separation supports JM503A as a novel species within the *Parvimonas* genus.

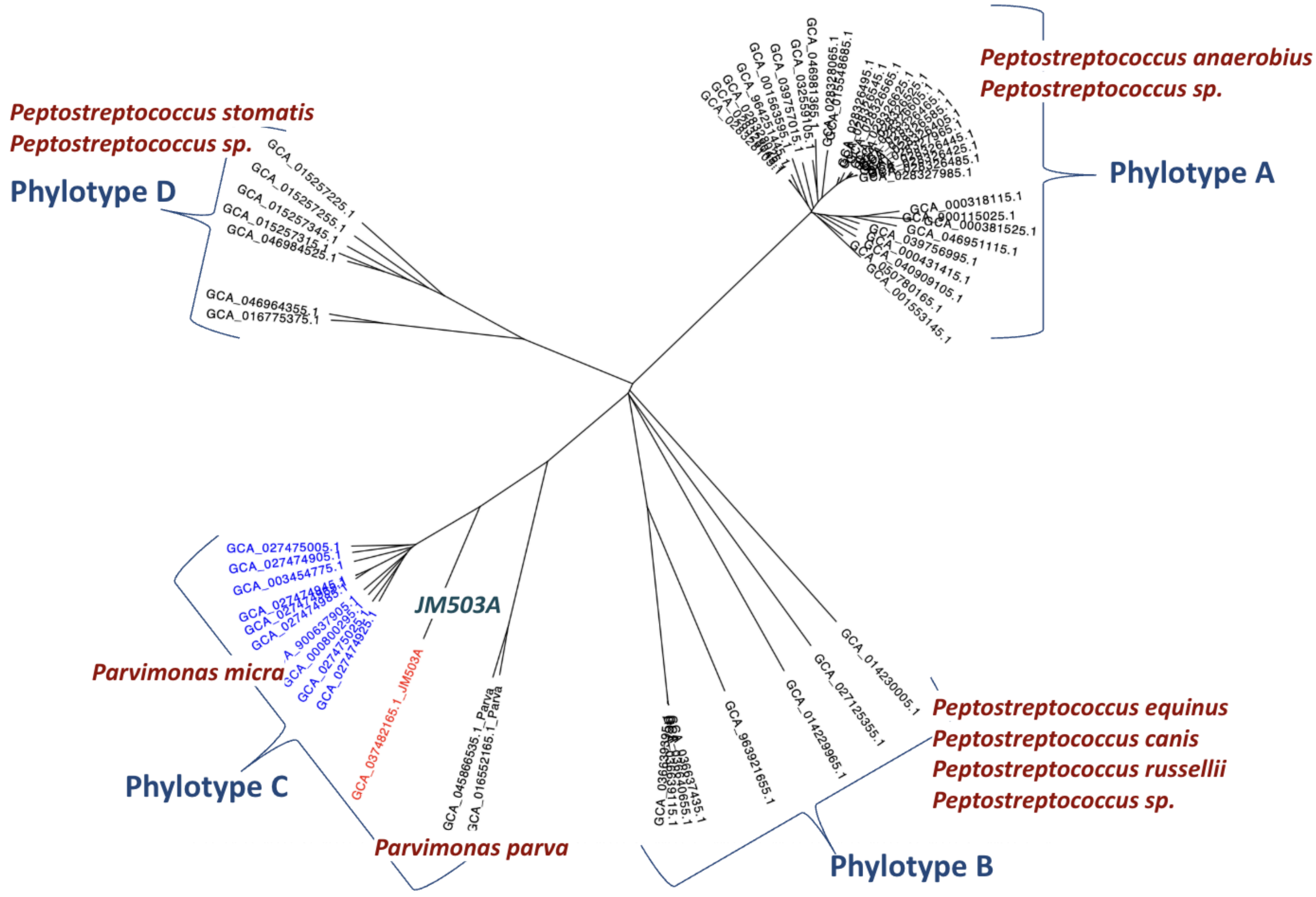

Supplementary Table 1. List of Parvimonas micra genomes used in this study. The table includes strain names and corresponding NCBI genome assembly accession numbers. These genomic sequences were retrieved from the NCBI Genome database and used for comparative genomic, phylogenetic, and taxonomic analyses throughout the study.

Sl. No.	Accession	Assembly Name	Organism Name	Strain	Annotation Name	Level	Release Date	Scaffolds
1	GCA_900637905.1	52756_G01	Parvimonas micra	NCTC11808	Annotation submitted by SC	Complete	43454	1
2	GCA_027474925.1	ASM2747492v1	Parvimonas micra	PM79KC-AC-2	NCBI Prokaryotic Genome Annotation Pipeline (PGAP)	Complete	44923	1
3	GCA_027475005.1	ASM2747500v1	Parvimonas micra	PM102KC-G-1	NCBI Prokaryotic Genome Annotation Pipeline (PGAP)	Complete	44923	1
4	GCA_027474905.1	ASM2747490v1	Parvimonas micra	PM79KC-G-1	NCBI Prokaryotic Genome Annotation Pipeline (PGAP)	Complete	44923	1
5	GCA_027474945.1	ASM2747494v1	Parvimonas micra	PM89KC-G-1	NCBI Prokaryotic Genome Annotation Pipeline (PGAP)	Complete	44923	1
6	GCA_027474965.1	ASM2747496v1	Parvimonas micra	PM89KC-G-2	NCBI Prokaryotic Genome Annotation Pipeline (PGAP)	Complete	44923	1
7	GCA_027474985.1	ASM2747498v1	Parvimonas micra	PM89KC-AC-1	NCBI Prokaryotic Genome Annotation Pipeline (PGAP)	Complete	44923	1
8	GCA_003454775.1	ASM345477v1	Parvimonas micra	KCOM 1037	NCBI Prokaryotic Genome Annotation Pipeline (PGAP)	Complete	43348	1
9	GCA_000800295.1	ASM80029v1	Parvimonas micra	KCOM 1535; ChDC B708	NCBI Prokaryotic Genome Annotation Pipeline (PGAP)	Complete	41982	1
10	GCA_037482165.1	ASM3748216v1	Parvimonas micra	JM503A	NCBI Prokaryotic Genome Annotation Pipeline (PGAP)	Complete	45375	1
11	GCA_027475025.1	ASM2747502v1	Parvimonas micra	PM114KC-AC-1	NCBI Prokaryotic Genome Annotation Pipeline (PGAP)	Complete	44923	1

Supplementary Table 2. Summary of RNA features identified in the genomes of *Parvimonas micra* strains analyzed in this study. The table includes the total number of RNA elements detected per genome, with breakdowns for tRNA, rRNA, and other RNA types.

GenBank / RefSeq	NCTC11808	PM79KC-AC-2	PM102KC-G-1	PM79KC-G-1	PM89KC-G-1	PM89KC-G-2	PM89KC-AC-1	KCOM 1037	KCOM 1535	JM503A	PM114KC-AC-1
repeat_region	0	1	1	2	2	1	3	1	1	1	1
tRNA	41	41	43	40	43	43	43	41	41	42	43
crispr_repeat	0	0	0	0	0	0	0	0	0	0	0
crispr_spacer	0	0	0	0	0	0	0	0	0	0	0
misc_RNA	0	0	0	0	0	0	0	0	0	0	0
rRNA	12	10	10	10	10	10	10	10	15	10	10
misc_binding	0	3	3	3	3	3	2	0	1	3	3
regulatory	0	3	3	3	3	3	3	2	0	3	3
crispr_array	0	0	0	0	0	0	0	0	0	0	0
misc_feature	0	1	1	1	1	1	1	0	11	1	1
ncRNA	0	2	2	2	2	2	2	2	1	2	2
tmRNA	1	1	1	1	1	1	1	1	0	1	1
Pseudogene	0	0	0	0	0	0	0	44	55	0	0

Supplementary Table 3. Phenotypic characteristics of the 11 *Parvimonas micra* strains analyzed in this study. NA - Not Available

Strain	Cell shape & size	Gram stain	Oxygen requirement	Motility	Spore formation	Catalase	Indole	Urease	Saccharolytic	Optimal Temperature	Fermentation Products	Natural competence
NCTC11808 (type)	Coccus, 0.3–0.7 µm, pairs/short chains	Positive	Obligate anaerobe	Non-motile	No	–ve	–ve	–ve	+ve	37°C	Acetate, small lactate/succinate	NA
PM79KC-AC-2	Coccus, 0.3–0.7 µm, pairs/short chains	Positive	Obligate anaerobe	Non-motile	No	–ve	–ve	–ve	+ve	37°C	acetate, butyrate, and propionate	NA
PM102KC-G-1	Coccus, 0.3–0.7 µm, pairs/short chains	Positive	Obligate anaerobe	Non-motile	No	–ve	–ve	–ve	+ve	37°C	acetate, butyrate, and propionate	NA
PM79KC-G-1	Coccus, 0.3–0.7 µm, pairs/short chains	Positive	Obligate anaerobe	Non-motile	No	–ve	–ve	–ve	+ve	37°C	acetate, butyrate, and propionate	NA
PM89KC-G-1	Coccus, 0.3–0.7 µm, pairs/short chains	Positive	Obligate anaerobe	Non-motile	No	–ve	–ve	–ve	+ve	37°C	acetate, butyrate, and propionate	NA
PM89KC-G-2	Coccus, 0.3–0.7 µm, pairs/short chains	Positive	Obligate anaerobe	Non-motile	No	–ve	–ve	–ve	+ve	37°C	acetate, butyrate, and propionate	NA
PM89KC-AC-1	Coccus, 0.3–0.7 µm, pairs/short chains	Positive	Obligate anaerobe	Non-motile	No	–ve	–ve	–ve	+ve	37°C	acetate, butyrate, and propionate	NA
KCOM 1037	Coccus, 0.3–0.7 µm, pairs/short chains	Positive	Obligate anaerobe	Non-motile	No	–ve	–ve	–ve	+ve	37°C	acetate, butyrate, and propionate	NA
KCOM 1535	Coccus, 0.3–0.7 µm, pairs/short chains	Positive	Obligate anaerobe	Non-motile	No	–ve	–ve	–ve	+ve	37°C	acetate, butyrate, and propionate	NA
JM503A	Coccus, 0.3–0.7 µm, pairs/short chains	Positive	Facultative anaerobe	Non-motile	No	–ve	–ve	–ve	+ve	37°C	NA	✓ high-level competence
PM114KC-AC-1	Coccus, 0.3–0.7 µm, pairs/short chains	Positive	Obligate anaerobe	Non-motile	No	–ve	–ve	–ve	+ve	37°C	acetate, butyrate, and propionate	NA

Supplementary Table 4. 16S rRNA gene BLAST results for strain JM503A. The table lists the closest matches identified, which were primarily partial sequences assigned to *Parvimonas* oral taxa. No highly similar full-length 16S rRNA sequences corresponding to complete *Parvimonas micra* genomes (highlighted in yellow box) were detected.

Description	Scientific Name	Max Score	Total Score	Query Cover	E value	Per. ident	Acc. Len	Accession
Parvimonas micra strain JM503A chromosome, complete genome	Parvimonas micra	2819	8413	100%	0	100	1549037	CP148048.1
Parvimonas micra canine oral taxon 076 clone OC026 16S ribosomal RNA gene, partial sequence	Parvimonas micra	2763	2763	99%	0	99.54	1517	JN713239.1
Parvimonas sp. oral taxon 110 clone HE064 16S ribosomal RNA gene, partial sequence	Parvimonas sp. oral taxon 110	2761	2761	98%	0	99.87	1501	GQ422714.1
Peptostreptococcus sp. oral clone HE064 16S ribosomal RNA gene, partial sequence	Peptostreptococcus sp. oral clone HE064	2750	2750	98%	0	99.73	1502	DQ087188.1
Parvimonas sp. oral taxon 110 16S ribosomal RNA gene, partial sequence	Parvimonas sp. oral taxon 110 str. F0139	2737	2737	98%	0	99.6	1500	GU470891.1
Uncultured bacterium partial 16S rRNA gene, clone 66q_15	uncultured bacterium	2726	2726	99%	0	99.14	1515	HE681293.1
Parvimonas sp. G1425 chromosome, complete genome	Parvimonas sp. G1425	2697	8043	100%	0	98.56	1501266	CP189843.1
Parvimonas sp. strain G1967 16S ribosomal RNA gene, partial sequence	Parvimonas sp.	2686	2686	100%	0	98.49	1523	PQ821450.1
Uncultured bacterium partial 16S rRNA gene, clone MA02H07	uncultured bacterium	2684	2684	97%	0	99.46	1476	FM873693.1
Uncultured bacterium partial 16S rRNA gene, clone MA01H11	uncultured bacterium	2684	2684	97%	0	99.46	1477	FM873640.1
Parvimonas sp. G1604 chromosome, complete genome	Parvimonas sp. G1604	2675	7980	100%	0	98.3	1450702	CP189844.1
Parvimonas micra strain KCOM 1037 chromosome, complete genome	Parvimonas micra	2671	7980	100%	0	98.29	1661863	CP031971.1
Uncultured bacterium partial 16S rRNA gene, clone 108_30	uncultured bacterium	2671	2671	99%	0	98.48	1515	HE681344.1

Parvimonas sp. strain G1425 16S ribosomal RNA gene, partial	Parvimonas sp.	2669	2669	100%	0	98.36	1523	PQ809779.1
Parvimonas micra strain PM89KC-AC-1 chromosome, complete genome	Parvimonas micra	2665	7991	100%	0	98.23	1663572	CP101411.1
Parvimonas micra strain PM89KC-G-2 chromosome, complete	Parvimonas micra	2665	7991	100%	0	98.23	1676568	CP101410.1
Parvimonas micra strain PM89KC-G-1 chromosome, complete genome	Parvimonas micra	2665	7991	100%	0	98.23	1676768	CP101409.1
Parvimonas sp. strain G1641 16S ribosomal RNA gene, partial sequence	Parvimonas sp.	2662	2662	100%	0	98.23	1523	PQ821452.1
Parvimonas sp. canine oral taxon 102 clone PO053 16S ribosomal RNA gene, partial sequence	Parvimonas sp. canine oral taxon 102	2658	2658	99%	0	98.29	1517	JN713266.1
Parvimonas micra strain PM79KC-G-1 chromosome, complete genome	Parvimonas micra	2658	7967	100%	0	98.1	1678242	CP101407.1
Parvimonas sp. strain KCOM 1749 (= ChDC B443) 16S ribosomal RNA gene, partial sequence	Parvimonas sp.	2658	2658	95%	0	99.79	1448	MT299685.1
Parvimonas micra strain PM79KC-AC-2 chromosome, complete genome	Parvimonas micra	2654	7947	100%	0	98.1	1755474	CP101408.1
Parvimonas micra strain PM102KC-G-1 chromosome, complete genome	Parvimonas micra	2654	7964	100%	0	98.1	1733605	CP101412.1
Parvimonas micra strain KCOM 1535, complete genome	Parvimonas micra	2654	7964	100%	0	98.1	1627009	CP009761.1
Parvimonas micra strain PM114KC-AC-1 chromosome, complete genome	Parvimonas micra	2654	7964	100%	0	98.1	1532745	CP101413.1
Parvimonas micra strain NCTC11808 genome assembly, chromosome: 1	Parvimonas micra	2654	7964	100%	0	98.1	1677398	LR134472.1
Parvimonas sp. strain KCOM 1630 (= ChDC B136) 16S ribosomal RNA gene, partial sequence	Parvimonas sp.	2652	2652	95%	0	99.72	1448	MT271919.1

Supplementary Table 5. Whole-genome BLAST comparison results of *Parvimonas micra* strain JM503A with other complete *P. micra* genomes. The table presents pairwise nucleotide identity percentages, total scores, and other alignment statistics.

Description	Scientific Name	Max Score	Total Score	Query Cover	E value	Per. ident	Acc. Len	Accession
Parvimonas micra strain JM503A chromosome, complete	Parvimonas micra	19324	20697	100%	0	100	1549037	CP148048.1
Parvimonas micra strain PM102KC-G-1 chromosome, complete genome	Parvimonas micra	15786	18844	100%	0	94.99	1733605	CP101412.1
Parvimonas micra strain PM79KC-G-1 chromosome, complete genome	Parvimonas micra	15777	18839	100%	0	94.39	1678242	CP101407.1
Parvimonas micra strain PM79KC-AC-2 chromosome, complete genome	Parvimonas micra	15732	18268	98%	0	95.19	1755474	CP101408.1
Parvimonas micra strain KCOM 1535, complete genome	Parvimonas micra	15703	18162	100%	0	95.15	1627009	CP009761.1
Parvimonas micra strain KCOM 1037 chromosome, complete	Parvimonas micra	15620	17677	100%	0	94.69	1661863	CP031971.1
Parvimonas micra strain PM114KC-AC-1 chromosome, complete genome	Parvimonas micra	15542	18055	98%	0	94.85	1532745	CP101413.1
Parvimonas micra strain PM89KC-G-2 chromosome, complete genome	Parvimonas micra	15536	17970	100%	0	93.95	1676568	CP101410.1
Parvimonas micra strain PM89KC-G-1 chromosome, complete genome	Parvimonas micra	15536	17970	100%	0	93.95	1676768	CP101409.1
Parvimonas micra strain NCTC11808 genome assembly, complete genome	Parvimonas micra	15527	17738	100%	0	94.82	1677398	LR134472.1
Parvimonas micra strain PM89KC-AC-1 chromosome, complete genome	Parvimonas micra	15509	17893	97%	0	94.79	1663572	CP101411.1
Parvimonas sp. G1425 chromosome, complete genome	Parvimonas sp. G1425	14772	16416	98%	0	92.66	1501266	CP189843.1
Parvimonas sp. G1604 chromosome, complete genome	Parvimonas sp. G1604	13380	15321	98%	0	90.94	1450702	CP189844.1

Supplementary Table 6. A heatmap contingency table was created using Molbiotools software to display pairwise intersections of accessory virulence genes across 11 *P. micra* strains, represented as a fully symmetrical matrix color-coded by intersection size. The copy number, ranging from 5 to 53, is indicated by a gradient from yellow to white.

Shared VFs	NCTC11808	PM79KC-AC-2	PM102KC-G-1	PM79KC-G-1	PM89KC-G-1	PM89KC-G-2	PM89KC-AC-1	KCOM 1037	KCOM 1535	JM503A	PM114KC-AC-1
**NCTC11808**	25	13	15	12	15	14	15	19	14	9	5
**PM79KC-AC-2**	13	30	20	14	19	18	19	21	18	8	5
**PM102KC-G-1**	15	20	51	31	28	27	28	33	23	15	3
**PM79KC-G-1**	12	14	31	44	23	22	25	25	15	15	5
**PM89KC-G-1**	15	19	28	23	52	47	51	26	24	17	5
**PM89KC-G-2**	14	18	27	22	47	47	46	25	24	16	5
**PM89KC-AC-1**	15	19	28	25	51	46	53	26	24	18	5
**KCOM 1037**	19	21	33	25	26	25	26	53	18	13	11
**KCOM 1535**	14	18	23	15	24	24	24	18	35	8	5
**JM503A**	9	8	15	15	17	16	18	13	8	29	7
**PM114KC-AC-1**	5	5	3	5	5	5	5	11	5	7	15

Supplementary Table 7. A comparison of the number of CAZymes across 11 *P. micra* strains revealed intragenomic variation among the CE, GH, and GT families. Variations among the strains, relative to other strains, were highlighted in blue, while the absence of specific families was indicated in pink.

Sl. No.	Assembly Accession	Organism Infraspecific Names Strain	CE Family	GH Family		GT Family					
			CE4	GH23	GH73	GT119	GT134	GT2	GT28	GT4	GT51
1	GCA_900637905.1	NCTC11808	1	0	1	3	1	2	2	1	1
2	GCA_027474925.1	PM79KC-AC-2	0	1	1	3	1	2	2	2	1
3	GCA_027475005.1	PM102KC-G-1	1	0	1	3	1	2	2	2	1
4	GCA_027474905.1	PM79KC-G-1	0	1	1	3	1	2	2	2	1
5	GCA_027474945.1	PM89KC-G-1	0	1	1	3	1	2	2	2	1
6	GCA_027474965.1	PM89KC-G-2	0	1	1	3	1	2	2	2	1
7	GCA_027474985.1	PM89KC-AC-1	0	1	1	3	1	2	2	2	1
8	GCA_003454775.1	KCOM 1037	2	1	1	3	0	2	2	1	1
9	GCA_000800295.1	KCOM 1535; ChDC B708	1	1	1	3	1	2	2	1	1
10	GCA_037482165.1	JM503A	1	1	1	3	1	2	2	2	1
11	GCA_027475025.1	PM114KC-AC-1	0	1	1	3	1	2	2	2	1

Enzymes abbreviated based on CAZyme classification: GH: Glycoside Hydrolase; CE: Carbohydrate Esterase; GT: Glycosyl Transferase
